# Photosynthetic and transcriptome responses to fluctuating light in *Arabidopsis thylakoid* ion transport triple mutant

**DOI:** 10.1002/pld3.534

**Published:** 2023-10-25

**Authors:** Peter J. Gollan, Steffen Grebe, Lena Roling, Bernhard Grimm, Cornelia Spetea, Eva‐Mari Aro

**Affiliations:** ^1^ Department of Life Technologies, Molecular Plant Biology University of Turku Turku Finland; ^2^ Institute of Biology/Plant Physiology Humboldt‐Universität zu Berlin Berlin Germany; ^3^ Department of Biological and Environmental Sciences University of Gothenburg Gothenburg Sweden; ^4^ Present address: Optics of Photosynthesis Laboratory, Institute for Atmospheric and Earth System Research (INAR)/Forest Sciences, Viikki Plant Science Center (ViPS) University of Helsinki Helsinki Finland

## Abstract

Fluctuating light intensity challenges fluent photosynthetic electron transport in plants, inducing photoprotection while diminishing carbon assimilation and growth, and also influencing photosynthetic signaling for regulation of gene expression. Here, we employed in vivo chlorophyll‐*a* fluorescence and P700 difference absorption measurements to demonstrate the enhancement of photoprotective energy dissipation of both photosystems in wild‐type *Arabidopsis thaliana* after 6 h exposure to fluctuating light as compared with constant light conditions. This acclimation response to fluctuating light was hampered in a triple mutant lacking the thylakoid ion transport proteins KEA3, VCCN1, and CLCe, leading to photoinhibition of photosystem I. Transcriptome analysis revealed upregulation of genes involved in biotic stress and defense responses in both genotypes after exposure to fluctuating as compared with constant light, yet these responses were demonstrated to be largely upregulated in triple mutant already under constant light conditions compared with wild type. The current study illustrates the rapid acclimation of plants to fluctuating light, including photosynthetic, transcriptomic, and metabolic adjustments, and highlights the connection among thylakoid ion transport, photosynthetic energy balance, and cell signaling.

## 1 INTRODUCTION

Plants use sunlight in the light reactions of photosynthesis to drive electron transport reactions in the thylakoid membrane of chloroplasts. The linear electron transport from photosystem II (PSII; P680) via cytochrome *b*_*6*_ *f* complex (Cytbf) toward photosystem I (PSI; P700) ultimately produces reducing power (NADPH) in the chloroplast stroma and is coupled to H^+^ translocation into the thylakoid lumen. This leads to the build‐up of a proton motive force (PMF) across the thylakoid membrane, composed of a pH (ΔpH) and an electric gradient (ΔΨ), driving ATP production at the ATP synthase. Together, NADPH and ATP are used for CO_2_ fixation and other metabolic processes within the chloroplast. To grow and thrive in natural environments where sunlight intensity quickly and extensively fluctuates, for example, due to intermittent cloud cover and sun flecks (Kaiser et al., 2018), plants have evolved different light acclimation responses (Gjindali et al., 2021; Long et al., 2022). These responses are highly dependent on the light environment during plant growth as well as the frequency, duration, and intensity of fluctuating light (FL) conditions (Alter et al., 2012; Niu et al., 2023; von Bismarck et al., 2023; Yin & Johnson, 2000). Short‐term responses also include photoprotective mechanisms (Allahverdiyeva et al., 2015) that are both rapidly inducible to prevent over‐excitation during high light and rapidly reversible to ensure sufficient photochemistry under low light. The most flexible photoprotective mechanisms in angiosperms are triggered by enhanced acidification of the thylakoid lumen. Increase in ΔpH triggers the regulated non‐photochemical quenching (NPQ), which consists of mechanisms to dissipate excess light energy as heat (Ruban, 2016). These mechanisms are enhanced by the lumenal protonation of the PSBS protein and by the low pH‐dependent interconversion of violaxanthin (V) to zeaxanthin (Z) in the xanthophyll cycle, mediated by violaxanthin de‐epoxidase (VDE) and zeaxanthin epoxidase (ZE). Furthermore, lumen acidification slows H^+^‐coupled electron transport through the Cytbf known as photosynthetic control (Tikhonov, 2014). This, in turn, leads to accumulation of oxidized PSI (P700^+^), which in itself is capable of harmlessly dissipating excess energy as heat (Schlodder et al., 2005).

Lumen pH‐dependent photoprotection is modulated by the influx/efflux of H^+^ and other ions (K^+^, Mg^2+^, and Cl^−^) through thylakoid ion channels and transporters. Ion exchange across thylakoid membranes directly modifies the ΔΨ component of the PMF and also adjusts the ΔpH, thereby integrating electron transport, NPQ, and ATP synthesis (Spetea et al., 2017). Rapid acidification of the lumen and subsequent induction of NPQ under high light requires influx of Cl^−^ counter‐ions through the voltage‐dependent chloride channel VCCN1, which leads to a decreased ΔΨ and consequently an increased ΔpH (Duan et al., 2016; Herdean et al., 2016). A similar function through the efflux of K^+^ co‐ions has been reported for the two‐pore potassium channel TPK3 (Carraretto et al., 2013), although its relevance for the regulation of photosynthesis is still debated (Höhner et al., 2019). The Cl^−^ channel/transporter CLCe is suggested to regulate thylakoid Cl^−^ homeostasis, ATP synthase activity, and electron transport during low light (Dukic et al., 2022; Herdean et al., 2016). The major pathway for H^+^ efflux from the lumen is through the ATP synthase complex, which is known to impact the formation and relaxation of ΔpH‐dependent NPQ (Kanazawa et al., 2017), whereas additional H^+^ efflux through the K^+^/H^+^ antiporter KEA3 accelerates NPQ downregulation after high light phases by decreasing ΔpH and increasing ΔΨ component of the PMF (Armbruster et al., 2014). VCCN1, CLCe, and KEA3 are known to function independently to adjust photosynthesis in response to changes in light intensities (Dukic et al., 2019; Li et al., 2021; von Bismarck et al., 2023).

Photoprotective mechanisms decrease the efficiency of electron transport and CO_2_ assimilation (Long et al., 2022; Murchie & Ruban, 2020) and impact the generation of reactive oxygen species (ROS) and other signals in the chloroplast, which modulate the expression of chloroplastic and nuclear genes (Gollan & Aro, 2020). ROS are produced at multiple sites inside the chloroplasts (Foyer & Hanke, 2022; Khorobrykh et al., 2020) and link photosynthesis to the inhibition of photosystems (Lima‐Melo et al., 2021; Zavafer & Mancilla, 2021), oxidation of lipids and carotenoids (Farmer & Mueller, 2013; Havaux, 2014), and the activity of many redox‐sensitive enzymes (Foyer & Hanke, 2022).

In the current study, we explored the interactions between energy balance in photosynthetic light reactions, global gene expression, and metabolism in *Arabidopsis thaliana* wild type (WT) and the triple mutant lacking KEA3, VCCN1, and CLCe (Dukic et al., 2019) exposed to 6 h FL and constant light (CL) treatments. FL conditions greatly enhanced photoprotective energy dissipation of both photosystems, albeit with altered kinetics leading to moderate PSI photoinhibition in *kvc*. FL‐induced gene expression changes in WT were associated particularly with secondary metabolism and biotic stress, whereas in *kvc*, the corresponding upregulation of biotic stress gene expression occurred already during growth under CL conditions, suggesting an indirect link between thylakoid ion channels/transporters and biotic stress response signaling.

## 2 MATERIALS AND METHODS

### 2.1 Plant growth and treatments

*Arabidopsis thaliana* Columbia‐0 (WT) plants and the *kea3‐1vccn1‐1clce‐2* (*kvc*) triple loss‐of‐function mutant along with respective single mutants *clce*, *vccn1*, and *kea3* (Dukic et al., 2019) were grown for 5 weeks in soil in short‐day conditions (8 h light/16 h darkness) at 23°C and 50% humidity under 100 μmol photons m^−2^ s^−1^ and watered three times a week with tap water. FL treatments were carried out using an LED array (Heliospectra, Sweden) set to alternate between low light (LL, 50 μmol photons m^−2^ s^−1^ for 4 min) and high light (HL, 500 μmol photons m^−2^ s^−1^ for 1 min). Five‐week‐old plants were treated with FL for 6 h. Control plants were exposed to CL at 100 μmol photons m^−2^ s^−1^ under the same LED array for 6 h. Plants were shifted to CL or FL conditions at the beginning of the photoperiod so that sample collection was performed toward the end of the photoperiod. Sample collection for all experiments was performed at the same time of day to minimize effects on photosynthetic performance and gene expression (Schneider et al., 2019). Long‐term FL effects on thylakoid protein abundances in WT and *kvc* were additionally investigated from plants grown under an FL regime for 6 weeks (same conditions as above).

### 2.2 In vivo chlorophyll‐*a* fluorescence and P700 difference absorption measurements

Simultaneous in vivo chlorophyll *a* fluorescence and P700 difference absorption measurements were performed from each genotype using a Dual‐PAM 100 (Walz, Germany) on mature leaves of CL‐ and FL‐treated plants. Measurements were performed with fluorescence measuring light intensity below <1.0‐μmol photons m^−2^ s^−1^ and maximum P700 measuring light intensity. Red light (635 nm) was used for actinic illumination for all measurements, including saturating pulses (SP, 700 ms, 8000 μmol photons m^−2^ s^−1^). Far‐red light (720 nm) intensity was set to 130 μmol photons m^−2^ s^−1^. PSII quantum yields and parameters were calculated as follows (Hendrickson et al., 2004; Kramer et al., 2004): maximum quantum yield of PSII photochemistry, *F*_*v*_/*F*_*m*_ = (*F*_*m*_ − *F*_0_)/*F*_*m*_; effective quantum yield of PSII photochemistry *Y*_*II*_ = (*F*_*m*_′ − *F*)/*F*_*m*_′; quantum yield of non‐regulated energy dissipation, *Y*_*NO*_ = *F*/*F*_*m*_; quantum yield of regulated NPQ, *Y*_*NPQ*_ = (*F*/*F*_*m*_′) − (*F*/*F*_*m*_); rate constant of NPQ, NPQ = (*F*_*m*_ − *F*_*m*_′)/*F*_*m*_′; and photochemical quenching parameter estimating the fraction of open PSII centers, *qL* = ((*F*_*m*_′ − *F*)/(*F*_*m*_′ − *F*_0_′)) × (*F*_0_′/*F*), with *F*_0_′ estimated according to Oxborough and Baker (1997). PSI quantum yields were calculated as follows (Klughammer & Schreiber, 2008): effective quantum yield of photochemistry in PSI, *Y*_*I*_ = (Δ*P*_*m*_′ − Δ*P*)/Δ*P*_*m*_; yield of PSI acceptor‐side limitation, *Y*_*NA*_ = (Δ*P*_*m*_ − Δ*P*_*m*_′)/Δ*P*_*m*_; yield of PSI donor‐side limitation, *Y*_*ND*_ = Δ*P*/Δ*P*_*m*_.

After CL or FL treatment, plants were dark acclimated for 30 min to determine minimum (*F*_0_) and maximum (*F*_*m*_) fluorescence directly with an SP, whereas Δ*P*_*m*_ was determined with an SP after additional 10 s of far‐red pre‐illumination and used for normalization of fluorescence and P700 traces as well as calculation of PSII and PSI parameters during separate experiments without dark acclimation. In these experiments, plants were subjected to a simulated FL regime with SPs at different time points throughout the measurement. For these measurements, *F*_0_ was estimated from fluorescence level during 10s far‐red illumination of the Δ*P*_*m*_ determination at the end of each experiment. This allowed estimation of *F*_*m*_ from *F*_0_ under far‐red light by using a rearranged PSII quantum yield equation *F*_*m*_ = *F*_0_/(1 − (*F*_*v*_/*F*_*m*_)) (Genty et al., 1989) and average *F*_*v*_/*F*_*m*_ from separate measurements after 30‐min dark acclimation of leaves (see above). This approach exploits oxidation of electron transport chain through the preferential excitation of PSI with far‐red light, which decreases *F* to the *F*_0_ level in the absence of PSII photoinhibition. The validity of this approach was confirmed by comparing the *F*_0_ level after 30‐min dark acclimation and after 10 s far‐red illumination, which showed no significant difference in fluorescence level (*T*‐test, *p* < 0.05, *n* = 4; Figure S1). A similar approach was used for normalizing fluorescence traces, whereas P700 traces needed to be corrected for signal drift assuming a constant steady‐state P700 oxidation level (P) at the end of each LL phase.

### 2.3 RNA isolation and transcriptome analysis

Mature leaves were harvested immediately after 6 h CL or FL treatment from four different plants of each genotype and snap frozen in liquid nitrogen. Total RNA was isolated using the Innuprep Plant RNA Kit (Analytik‐Jena, Germany). RNA libraries were prepared and sequenced on an Illumina HiSeq 2000 platform at BGI Genomics (China) and deposited in NCBI sequence read archive (PRJNA735049). Reads from RNA‐seq were aligned to the *A. thaliana* reference genome containing gene annotations described in TAIR10, using Stand NGS software v3.4 (Avadis, India). Gene expression was quantified and normalized to the median expression value of all genes using the DESeq package (R). Differential expression of genes with log2 fold change (log2 FC) ≥ 1 was determined by a two‐way ANOVA test. *P*‐values were adjusted for false discovery rate (FDR) using the Benjamini–Hochberg procedure.

Gene ontology (GO) enrichment analysis was performed using the Gene Ontology Online Resource (http://pantherdb.org/webservices/go/overrep.jsp) to identify biological processes GO terms that were statistically significantly enriched (FDR corrected *P* < .05) in gene lists of interest. Non‐redundant GO term lists were obtained by manually collapsing overlapping GO terms into a single term. Subcellular loci were predicted using the subcellular localization database (SUBA4; https://suba.live/).

### 2.4 Pigment analysis

For pigment extraction, 50 mg of leaf material from WT and *kvc* was ground in liquid nitrogen directly after 6 h CL and FL treatments. The pigments were extracted using alkaline acetone: 0.2 M NH_4_OH 9:1 (v/v) and incubation for 30 min at −20°C followed by centrifugation for 30 min (15,000 × *g*, 4°C). The HPLC analysis of the pigment‐containing supernatant was performed on Agilent LC systems (Agilent, USA) equipped with a DAD detector (440 nm, Agilent 1100 series) using a Prontosil column (200‐3‐C30, 3 μm; Bischoff Chromatography, Germany). For gradient elution, the composition of the mobile phase (A:B, v/v) with A = 90% acetonitrile, 10% H_2_O, 0.1% triethylamine, and B = 100% ethyl acetate was changed as follows: *t*_0min_ 100:0, *t*_1min_ 60:40, *t*_19min_ 0:100, *t*_20min_ 0:100, *t*_2.1–23min_ 100:0. The mobile phase flow rate was 1.6 mL min^−1^. Peaks were quantified using authentic standards.

### 2.5 Thylakoid protein analysis

Rosettes from 6‐week‐old WT and *kvc* grown under FL conditions were harvested for protein analysis. Thylakoid isolations, chlorophyll determination, gel electrophoresis, and western blotting were performed as described previously (Dukic et al., 2022). Proteins were recognized by specific antibodies against PsaB (1:3000, Agrisera), CP47 (1:3000, gift from Prof. Barbato), D1 (1:8000, gift from Prof. Barbato), LHCB2 (1:5000, Agrisera), CytF (1:5000 Agrisera), AtpF (1:10000, Agrisera), PSBS (1:5000, gift from Prof. Barbato), VDE (1:1000, gift from Prof. Åkerlund), and ZE (1:2000, Agrisera) and detected with horseradish peroxidase‐linked secondary antibody (Agrisera, Sweden) and Amersham ECL Western blotting detection reagents (GE Healthcare, USA).

### 2.6 Leaf starch content assay

Starch was isolated from mature leaves of 5‐week‐old WT and *kvc* plants subjected to 6 h CL or FL treatment. One hundred milligrams of leaf tissue was weighed and extracted three times by bead‐beating in 800 μL of methanol:chloroform:water (12:5:3, v/v). The insoluble pellet containing starch was quantified using a total starch assay kit (Megazyme, Ireland).

### 2.7 Cuticle permeability assay

A toluidine blue O (TBO) leaf drop assay was performed as described by Cui et al. (2019). Two mature leaves were harvested from each of eight individual WT and *kvc* that had been treated with either 6‐h CL or FL. Five‐micoliter drops of 0.05% TBO in acetate buffer (pH 4) were pipetted onto the adaxial surface of each leaf and incubated on wet paper towel for 2 h. Leaves were rinsed thoroughly with distilled water and photographed with a Canon DSLR camera. The extent of TBO infiltration was quantified using ImageJ (https://imagej.nih.gov/ij/) to count the total number of pixels within each TBO‐stained area.

## 3 RESULTS

### 3.1 Fluctuating light alters the kinetics of PSII and PSI energy distribution and leads to moderate PSI photoinhibition in *kvc*

To resolve the effect of FL on the regulation of energy distribution between photosystems, we compared WT and *kvc* plants treated for 6 h with either CL or FL via simultaneous measurements of chlorophyll‐*a* fluorescence of PSII (Fluo) and P700 difference absorption of PSI (P700) in two individual experiments.

**FIGURE 1:**
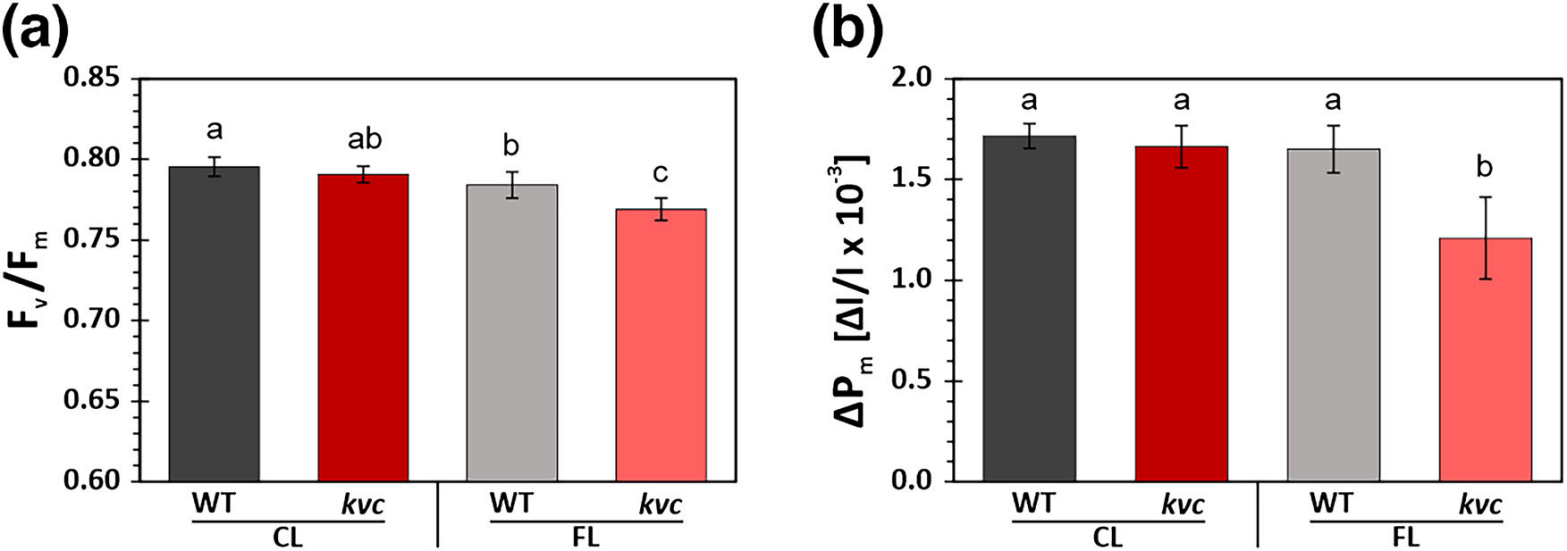
Maximal PSII quantum yield and maximal redox active PSI fraction of WT and *kvc* after CL and FL treatments. Estimation of (a) maximal quantum yield of photochemistry in PSII (*F*_*v*_/*F*_*m*_) and (b) maximal redox active fraction of PSI (Δ*P*_*m*_) of dark acclimated wild type (WT, gray) and *kvc* triple mutant (*kvc*, red) after treatment for 6 h with constant light (CL, 100 μmol photons m^−2^ s^−1^) or 6 h with fluctuating light (FL, 50 μmol photons m^−2^ s^−1^ for 4 min and 500 μmol photons m^−2^ s^−1^ for 1 min). Data represent mean with letters indicating statistically significant groups (ANOVA, Tukey‐HSD, *P* < 0.05, error bars denote SD, *n* = 10–12).

**FIGURE 2:**
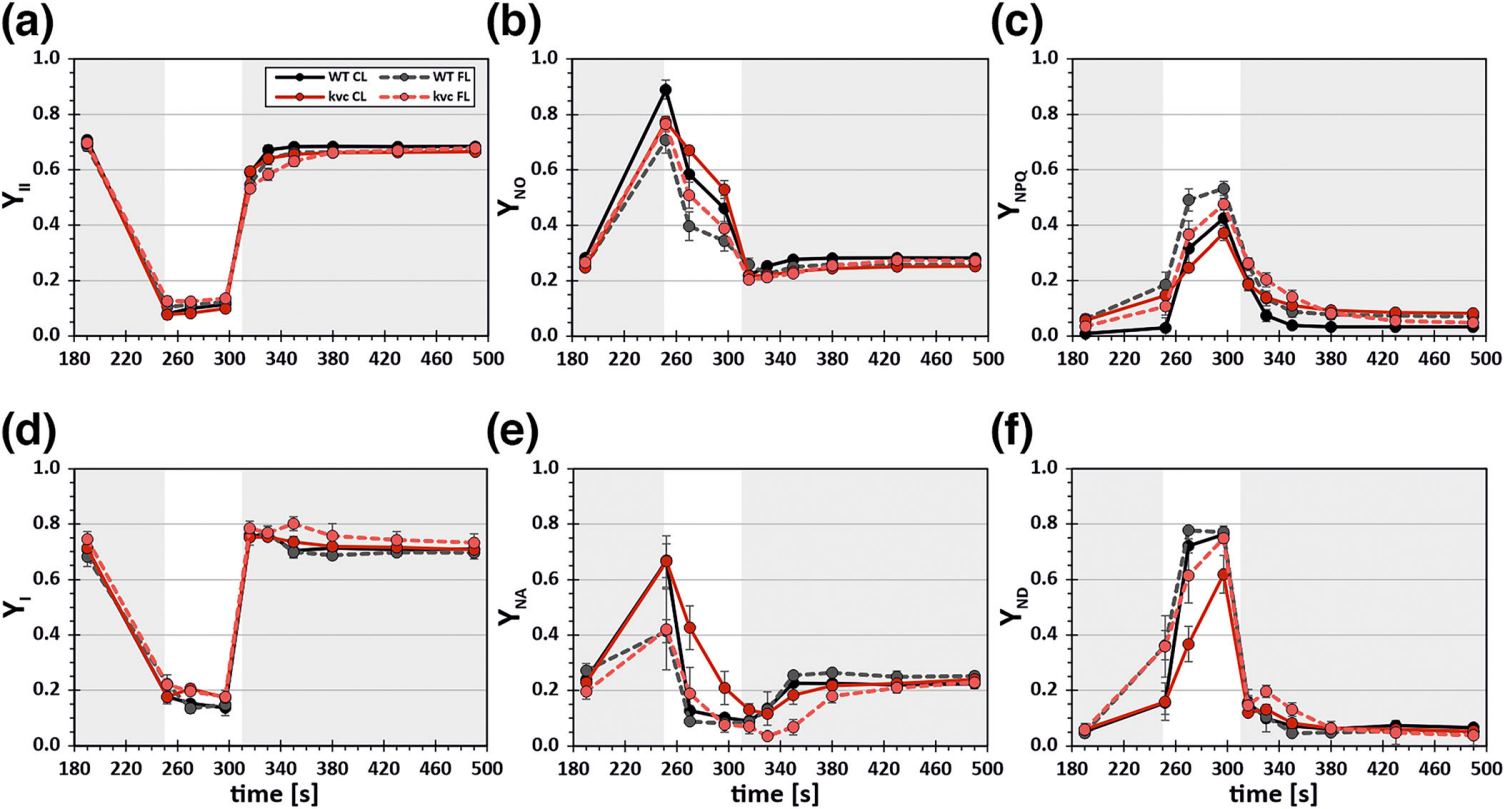
Changes in PSII and PSI quantum yields in WT and *kvc* after CL and FL treatments. Wild type (WT) and *kvc* triple mutant (*kvc*) plants were treated for 6 h with constant light (CL, 100 μmol photons m^−2^ s^−1^) or 6 h with fluctuating light (FL, 50‐μmol photons m^−2^ s^−1^ for 4 min and 500 μmol photons m^−2^ s^−1^ for 1 min) immediately prior to measurements with a Dual‐PAM 100 system and subjected to a single FL cycle without dark‐acclimation (for details, see Section 2). (a) Effective quantum yield of photochemistry in PSII (Y_II_); (b) quantum yield of non‐regulated energy dissipation (Y_NO_); (c) quantum yield of regulated non‐photochemical quenching (Y_NPQ_); (d) effective quantum yield of photochemistry in PSI (Y_I_); (e) yield of PSI acceptor‐side limitation (Y_NA_); (f) yield of PSI donor‐side limitation (Y_ND_). Data represents mean with error bars indicating standard deviation (*n* = 3–4).

In the first experiment, CL‐ and FL‐treated WT and *kvc* plants were dark‐acclimated to determine the influence of different light treatments on the maximal quantum yield of photochemistry in PSII (*F*_*v*_/*F*_*m*_, Figure 1a) and the maximal redox active fraction of PSI (Δ*P*_*m*_, Figure 1b). The FL treatment led to a small decrease (2%) in *F*_*v*_/*F*_*m*_ in both WT and *kvc* as compared with CL treatment, which was slightly more pronounced in *kvc*. In contrast, FL treatment had no significant effect on Δ*P*_*m*_ in WT, whereas it resulted in an average decrease of 27% in *kvc* as compared with CL conditions, suggesting a moderate amount of PSI photoinhibition in *kvc*. Similar results were also obtained for single mutants *clce*, *kea3*, and *vccn1*, which showed equal reduction of *F*_*v*_/*F*_*m*_ (Figure S2A) and slightly less pronounced reduction of Δ*P*_*m*_ (Figure S2B) compared with *kvc*, especially in *clce*.In the second experiment, WT and *kvc* plants were recorded during a single FL cycle by directly transferring plants to the Dual‐PAM‐100 without dark‐acclimation to maintain the corresponding 6 h CL‐ and FL‐acclimation states of the plants (see Section 2). Fluo and P700 traces revealed overall slower responses in *kvc* mutant as compared with WT, and FL treatment further altered Fluo and P700 kinetics (Figure S3), suggesting rapid changes in energy distribution within PSII and PSI, especially during HL and after shift from HL to LL phase. These changes were further investigated by including saturating pulses in the single FL cycle, which allowed calculation of PSII and PSI quantum yields (Figure 2). During the HL phase, a substantial decrease in effective quantum yield of PSII (Y_II_, Figure 2a) was accompanied by an initial strong increase in quantum yield of non‐regulated energy dissipation (Y_NO_, Figure 2b), which was successively replaced by an increase in quantum yield of regulated NPQ (Y_NPQ_) (Figure 2c). Upon transition to the second LL phase, all PSII quantum yields subsequently returned to their previous levels. Although the PSII quantum yields generally followed a similar response, substantial differences between WT and *kvc* upon the light treatment were evident. During HL, *kvc* showed a slower rise in Y_NPQ_ as compared with WT after CL treatment, whereas FL treatment systematically increased Y_NPQ_ in both WT and *kvc* as compared with CL conditions (Figure 2c). The lower Y_NPQ_ in *kvc* compared with WT did not lead to changes in Y_II_ during the HL phase, but rather to higher Y_NO_ in both CL and FL conditions (Figure 2a,b). Only during transition to the second LL phase, Y_II_ showed a slightly slower recovery to initial levels in *kvc* compared with WT, which matched the corresponding slower decline in Y_NPQ_. This effect on Y_NPQ_ was more pronounced in direct comparisons of the rate of NPQ in *kvc* with WT and was present regardless of different levels of NPQ after CL and FL treatments (Figure S4A). Additional comparison of the redox state of Q_A_ in PSII, reflecting open (oxidized) or closed (reduced) PSII centers via the photochemical quenching parameter qL, revealed similar responses in both genotypes and light treatments (Figure S4B). Here, the majority of PSII centers were closed during HL and rapidly re‐opened after transition to LL. However, during transition to the second LL phase, PSII centers were transiently more open in *kvc* after FL treatment as compared with WT and CL conditions.

Analogous to PSII, also PSI quantum yields in both genotypes (WT and *kvc*) and light treatments showed a similar general response. During the HL phase, this comprised a strongly diminished effective quantum yield of PSI (Y_I_, Figure 2d), which was caused by an initial increase in the yield of PSI acceptor‐side limitation (Y_NA_, Figure 2e) and successive replacement by an increase in yield of PSI donor‐side limitation (Y_ND_, Figure 2f). Upon transition to the second LL phase, all PSI yields ultimately returned back to initial levels, whereby Y_NA_ and Y_ND_ showed overall slower kinetics compared to Y_I_. However, in clear difference to WT, the *kvc* mutant showed a slower increase in Y_ND_ (Figure 2f) and a corresponding slower decline of Y_NA_ (Figure 2e) during HL after the CL treatment. FL treatment, on the other hand, led to a stronger initial increase in Y_ND_ and equivalent decrease of Y_NA_ both in WT and *kvc*, although upon transition to the second LL phase, *kvc* showed transiently lower Y_NA_ (Figure 2e) and correspondingly higher Y_ND_ (Figure 2f) compared with WT. Importantly, these corresponding changes of Y_ND_ and Y_NA_ did not substantially alter Y_I_, except for a transient increase in *kvc* after FL treatment during the HL‐LL transition (Figure 2d).

Single mutants *clce*, *kea3*, and *vccn1* showed equivalent responses of PSII and PSI quantum yields like WT and *kvc* from 6 h CL (Figure S5) and FL treatments (Figure S6) as detailed above, with a pronounced lower induction of Y_NPQ_ in *vccn1* during the HL phase and a prolonged induction of Y_NPQ_ in *kea3* during the HL‐LL transition, as previously described (Armbruster et al., 2014; Duan et al., 2016; Herdean et al., 2016).

In summary, the 6 h FL treatment led to stronger PSI than PSII photoinhibition in *kvc* and single mutants compared with WT. During the HL phase, in all genotypes, Y_II_ or Y_I_ were not differently affected in FL compared with CL conditions, but the HL phase led to complementary upregulation and downregulation of Y_NPQ_ and Y_NO_ as well as Y_ND_ and Y_NA_ of PSII and of PSI, respectively.

### 3.2 Thylakoid protein changes in long‐term FL growth conditions

To gain further insight into the regulation of photosynthetic energy balance on the protein level, we investigated changes in thylakoid protein abundances in plants grown under FL conditions, as short‐term light changes typically do not lead to major adjustments of photosynthetic protein contents (Kono & Terashima, 2014; Schöttler & Tóth, 2014) in contrast to long‐term acclimation (Flannery et al., 2021; Niedermaier et al., 2020). Short‐term changes in protein abundance of photosystems were especially not expected, due to the rapid repair of damaged PSII centers via D1 protein turnover (Aro et al., 1993; Järvi et al., 2015) and high stability of PSI reaction center proteins without repair, despite severe PSI photoinhibition (Kudoh & Sonoike, 2002; Lempiäinen et al., 2022; Tiwari et al., 2016; Zhang & Scheller, 2004). In *kvc*, the long‐term growth in FL conditions revealed a decrease in PSI (PsaB), ATP synthase (AtpF), and only a minor decrease in PSII (D1 and CP47) protein contents, whereas Cytbf (PetA), LHCII (LHCB2), and ZE contents remained similar, and PSBS together with VDE increased in comparison with WT (Figure S7).

### 3.3 Modulations in transcript accumulation of WT and *kvc* upon 6 h exposure to fluctuating light

**FIGURE 3:**
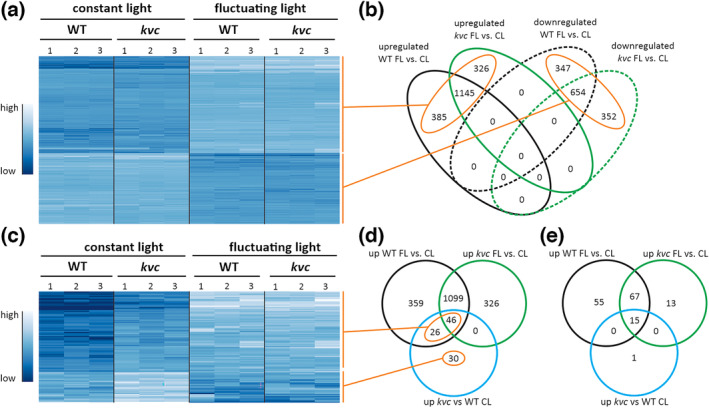
Expression pattern and comparisons of genes upregulated in *kvc* in relation to wild type. (a) Hierarchically clustered heatmap showing gene expression levels, normalized to median level expression, in three replicates of wild type (WT) and *kvc* samples exposed to 6 h constant light (CL) or 6 h fluctuating light (FL). Shown are all genes with differential expression in WT or *kvc* (DE; FC ≥ log2 2, FC ≤ log2–2) after FL exposure, in comparison with CL exposure. Gene expression level is shown according to color scale. (b) Venn diagram analysis of overlap between genes upregulated or downregulated in the FL vs. CL comparisons in each genotype. Orange circles indicate corresponding gene clusters shown in the heatmap (a). (c) Hierarchically clustered heatmap showing expression of 102 genes found to be upregulated in *kvc* under CL conditions, compared to WT under CL. Color range indicates normalized gene expression level. (d) Venn diagram illustrating the overlap between 102 genes upregulated in *kvc* vs. WT and those upregulated in WT FL vs. CL and *kvc* FL vs. CL (shown in b). Orange circles indicate corresponding gene clusters shown in the heatmap (c). (e) Venn diagram illustrating the overlap between 16 non‐redundant gene ontology (GO) terms found enriched in genes upregulated in *kvc* vs. WT and those found in WT FL vs. CL and *kvc* FL vs. CL. Full lists of genes and GO terms are included in Data S1 and S2.

In order to explore the effects of short‐term FL on gene expression, especially in the context of dynamic photosynthetic responses due to lack of ion channels/transporters, the transcriptomes of WT and the *kvc* mutant exposed for 6 h to either CL or FL were primarily analyzed. The expression of approximately 1500 genes was upregulated at least twofold (log2 FC ≥ 1) in the FL versus CL comparison in each genotype and 1145 of these genes were commonly upregulated in both genotypes (Figure 3a,b). Notably, KEA3, VCCN1, and CLCe were not differentially expressed (DE) above the established threshold when FL and CL treatments were compared in WT (Data S1). Similarly, the expression of other known K^+^, Cl^−^, and Mg^2+^ channels/transporters, as well as mechanosensitive and gated ion channels in the thylakoid and other membranes, were also not DE by FL treatment in either WT or *kvc* (Data S1).GO terms enriched in the FL‐upregulated genes of both WT and *kvc* included wax/fatty acid and terpene biosynthesis, and responses to hypoxia, herbivory, and biotic stress (Table 1). Around 1000 genes were downregulated by FL treatment, of which 654 genes were common for WT and *kvc* (Figure 3a,b). GO terms significantly enriched among downregulated genes included DNA replication and regulation of salicylic acid signaling (Table 1). Full lists of DE genes and GO terms can be accessed from Data S1 and S2.

**TABLE 1:** Gene ontology (GO) terms significantly enriched in upregulated or downregulated genes of WT and *kvc.*

GO term	GO term ID	Fold enrichment WT FL vs. CL	Fold enrichment *kvc* FL vs. CL	Fold enrichment *kvc* vs. WT CL
Wax biosynthesis	GO:0010025	7.8	6.1	n.s.
Response to hypoxia	GO:0001666	6.2	5.0	n.s.
Terpenoid metabolism	GO:0006721	3.3	3.6	n.s.
Response to herbivore	GO:0080027	1.9	8.0	n.s.
Response to biotic stimulus	GO:0009607	3.8	3.3	7.3
Defense response	GO:0006952	3.3	3.1	6.7
DNA replication	GO:0006260	9.5	5.0	n.s.
Regulation of SA signaling	GO:2000031	8.9	9.0	n.s.

*Note*: GO terms enriched in upregulated genes are colored light blue; GO terms enriched in downregulated genes are colored dark blue; n.s., not significant.

Comparison of the WT and *kvc* triple mutant transcriptomes revealed under CL conditions only around 140 DE genes, of which 102 were upregulated in *kvc*. After FL exposure, over 70% of these genes were also upregulated in WT plants, 45% were upregulated in *kvc* in the FL versus CL comparison, and around 30% were unresponsive to or downregulated by FL (Figure 3c,d). Intriguingly, these data indicate that a majority of genes with higher expression in *kvc* than in WT under CL conditions are actually part of the FL regulon. GO term analysis showed significant enrichment in the *kvc*‐upregulated genes for responses to biotic stimuli, including defensive responses to fungus and bacteria (Data S2), terms that were found upregulated by FL in both WT and *kvc* (Table 1). Comparative analysis showed almost complete overlap between *kvc‐*upregulated and FL‐upregulated GO terms of WT (Figure 3c and Data S2), further suggesting commonality between FL‐ and *kvc*‐responsive processes.

In‐depth transcriptome analysis revealed upregulation of secondary metabolic pathways in both WT and *kvc* genotypes after FL exposure. In particular, enzymes involved in the metabolism of tocopherol and carotenoids, and those involved in long‐chain fatty acid, wax, and suberin synthesis were upregulated (Table 2). Notably, nine genes encoding berberine bridge enzyme (BBE)‐like proteins were upregulated after FL treatment, corresponding to 75% of the BBE‐like enzymes with statistical significance in the current experiment (Table 2), and 35% of the entire BBE‐like enzyme family in Arabidopsis (Daniel et al., 2017). BBE‐like enzymes catalyze oxidation of various secondary metabolites, and some are involved in pathogen response (Benedetti et al., 2018). Several factors involved in synthesis and degradation of the terpenoid hormones abscisic acid (ABA) and gibberellic acid (GA) were DE by FL in both WT and *kvc*. Signaling activity of the fatty acid derivative hormone jasmonic acid (JA) appeared to be targeted by upregulation of several factors involved in JA degradation and turnover (Table 2).

**TABLE 2:** Genes demonstrating significantly differential expression between WT and *kvc* as induced by exposing the plants to FL conditions.

Name	AGI code	Description	log2FC FL vs. CL WT	log2FC FL vs. CL *kvc*	*P*‐value*
Terpenoid synthesis					
GGPPS7	AT2G18620	Terpene biosynthesis	2.3	1.7	0.000
SPS1	AT1G78510		1.2	1.2	0.000
SPS2	AT1G17050		1.1	1.3	0.000
TPS03	AT4G16740		3.3	2.4	0.002
TPS10	AT2G24210		2.8	1.9	0.010
GES	AT1G61120		3.9	1.0	0.003
CHY1	AT4G25700	Xanthophyll synthesis from β‐carotene	1.7	2.2	0.000
VTE1	AT4G32770	Tocopherol/quinone synthesis	1.3	1.2	0.001
PDS1	AT1G06570		1.3	0.9	0.004
CPT4/CTP7	AT5G58770	Polyprenol synthesis	1.1	0.7	0.000
Fatty acid, wax synthesis					
KCS1	AT1G01120	Long chain fatty acid synthesis/elongation	1.5	1.3	0.000
KCS2	AT1G04220		2.9	2.1	0.000
KCS3	AT1G07720		1.3	1.0	0.000
KCS5	AT1G25450		1.3	1.1	0.000
KCS6	AT1G68530		1.4	0.8	0.000
KCS8	AT2G15090		2.8	2.5	0.000
KCS12	AT2G28630		3.2	3.3	0.003
KCS19	AT5G04530		−2.8	−2.3	0.000
KCS20	AT5G43760		1.1	0.5	0.000
KCR2	AT1G24470		4.4	3.7	0.000
FABG	AT3G04000		1.6	1.6	.000
CER8 (LACS1)	AT2G47240		2.6	3.4	0.002
LACS3	AT1G64400		1.9	2.0	0.000
AT5G47330	AT5G47330		2.9	3.6	0.004
CER1	AT1G02205	Wax/suberin/cutin synthesis and secretion	1.9	0.6	0.002
CER3 (WAX2)	AT5G57800		1.1	1.2	0.000
FAR1	AT5G22500		1.7	1.1	0.008
WSD1	AT5G37300		4.4	3.3	0.000
WDS6	AT3G49210		2.1	2.0	0.000
WSD‐like	AT5G16350		−0.9	−0.2	0.013
MAH1	AT1G57750		3.1	2.4	0.000
Berberine bridge enzyme (BBE)‐like domain‐containing protein					
AtBBE3	AT1G26380	Oxidation of secondary metabolites	5.0	5.7	0.000
AtBBE6	AT1G26410		4.4	4.8	0.000
AtBBE7	AT1G26420		7.2	5.5	0.000
AtBBE8	AT1G30700		3.8	3.6	0.000
AtBBE9	AT1G30720		7.2	6.7	0.000
AtBBE11	AT1G30730		6.2	6.0	0.000
AtBBE18	AT4G20860		3.2	3.3	0.000
AtBBE22	AT4G20820		2.3	1.0**	0.000
AT5G44380	AT5G44380		2.2	2.0	0.004
Regulation of hormone signaling					
NCED3	AT3G14440	Abscisic acid synthesis/catabolism	1.2	1.0	0.041
NCED5	AT1G30100		1.5	0.6	0.000
CYP707A2	AT2G29090		1.8	1.6	0.000
CYP707A3	AT5G45340		4.8	1.7	0.000
ABA2	AT1G52340		−1.1	−0.7	0.012
GA2OX1	AT1G78440	Gibberellic acid oxygenase	1.2	1.8	0.012
GA2OX4	AT1G02400		4.8	4.6	0.000
GA2OX8	AT4G21200		0.9	2.1	0.004
GA3OX1	AT1G15550		2.4	2.8	0.000
GA3OX2	AT1G80340		1.7	2.4	0.014
GA20OX1	AT4G25420		−1.7	−1.7	0.000
GA20OX2	AT5G51810		1.7	1.5	0.003
JOX2	AT5G05600	Jasmonic acid oxygenase	2.1	0.3**	0.010
JOX3/JRG21	AT3G55970		3.6	1.6	0.010
JOX4	AT2G38240		4.2	3.7	0.000
CYP94B1	AT5G63450	Jasmonic acid‐Ile turnover	3.1	1.3	0.041
CYP94C1	AT2G27690		1.5	0.6	0.015
ILL5	AT1G51780		7.2	3.9**	0.000
OPR‐like	AT1G18020	12‐oxophytodienoic acid reduction, jasmonic acid synthesis	−1.9	−2.3	0.000
OPR‐like	AT1G09400		−1.3	−2.5	0.003
OPR‐like	AT1G17990		−2.2	−2.1	0.000
Abiotic stress‐related transcription regulators					
HSFA3	AT5G03720	Heat shock transcription factor	1.7	1.9	0.000
HSFA6A	AT5G43840		2.5	1.5	0.012
HSFA6B	AT3G22830		1.6	0.0	0.002
HSFA8	AT1G67970		1.4	1.0	0.000
HSFB2A	AT5G62020		1.3	0.8	0.003
DREB2A	AT5G05410	Dehydration‐responsive element‐binding protein	2.0	1.7	0.000
DREB2B	AT3G11020		1.2	1.1	0.011
DREB2C	AT2G40340		3.1	2.2	0.029
DREB2E	AT2G38340		2.6	1.6	0.009
ZAT10	AT1G27730	Zinc finger transcription factor	4.4	4.1	0.005
ZAT12	AT5G59820		5.1	4.1	0.000
ABI5	AT2G36270	Abscisic acid‐responsive transcription	1.4	−0.1	0.046
NAC046	AT3G04060	NAC domain‐containing regulator	2.7	0.7**	0.005
Photorespiration					
GOX1	AT3G14420	Glycolate/glyoxylate catabolism	1.0	1.1	0.000
AGT1	AT2G13360		1.1	1.1	0.000
AGT3	AT2G38400		1.6	1.9	0.000
GGT2	AT1G70580		1.2	1.4	0.000
GLPD2	AT2G26080		1.7	1.8	0.000
GLN1;1	AT5G37600		1.4	1.7	0.000

*Note*: Gene expression >2FC upregulated is colored light blue; >2FC downregulated is colored dark blue.

* Corrected *P*‐value using the Benjamini–Hochberg method.

** Upregulated >2FC in *kvc* vs. WT after CL exposure.

Expression of many abiotic stress‐related transcriptional regulators was substantially upregulated by FL (Table 2), including several heat shock factors (HSFs), dehydration‐responsive (DREB2) factors, and the zinc‐finger proteins ZAT10 and ZAT12. Notably, the ABA‐ and stress‐responsive transcription factor ABI5 was upregulated by FL in WT but was unresponsive to FL in *kvc* (Table 2). Other FL‐induced abiotic stress‐related factors included the cold‐responsive COR27, COR15A, and COR15B; DnaJ‐type protein chaperones; and the chloroplastic iron chaperones FER1, FER3, and FER4 (Data S1).

To investigate possible similarities of the FL regulon with other biotic and abiotic stresses, genes upregulated by FL in WT and *kvc* were compared with published transcriptomic data. This analysis showed only 11%–12% overlap with genes upregulated after 60‐min HL exposure (Figure S8; Gene Expression Omnibus [GEO] accession GSE94075; Crisp et al., 2017), whereas even lower overlap was observed with genes responsive to drought stress (GEO accession GSE65046; Bechtold et al., 2016; GEO accession GSE24177; Harb et al., 2010; not shown). On the other hand, around 40% of genes upregulated by flagellin peptide flg22, artificially triggering plant biotic response (GEO accession GSE5615; Qutob et al., 2006), were upregulated by FL in WT and/or the *kvc* mutant (Figure S8).

Taken together, these analyses revealed a strong upregulation in the expression of secondary metabolism and biotic stress‐responsive genes in both WT and *kvc*, demonstrating that FL induction of these genes is mostly independent of KEA3, VCCN1, and CLCe in Arabidopsis. This was further supported by largely similar transcriptome response to short‐term FL treatment of *kea3*, *vccn1*, and *clce* single mutants compared with both *kvc* and WT in upregulated GO‐terms (Table S1) and differentially expressed genes (Table S2). However, although transcriptional changes associated with biotic stress defense were induced by FL in WT, *kvc*, and single mutants (Table S1), these were specifically upregulated in *kvc* as well as *vccn1* and *clce* already under non‐stress CL conditions, suggesting a potentially additive influence of altered thylakoid Cl^−^ ion‐homeostasis on the biotic stress response.

### 3.4 FL exposure impacts the xanthophyll cycle pigment and abundance of a chlorophyll precursor

**FIGURE 4:**
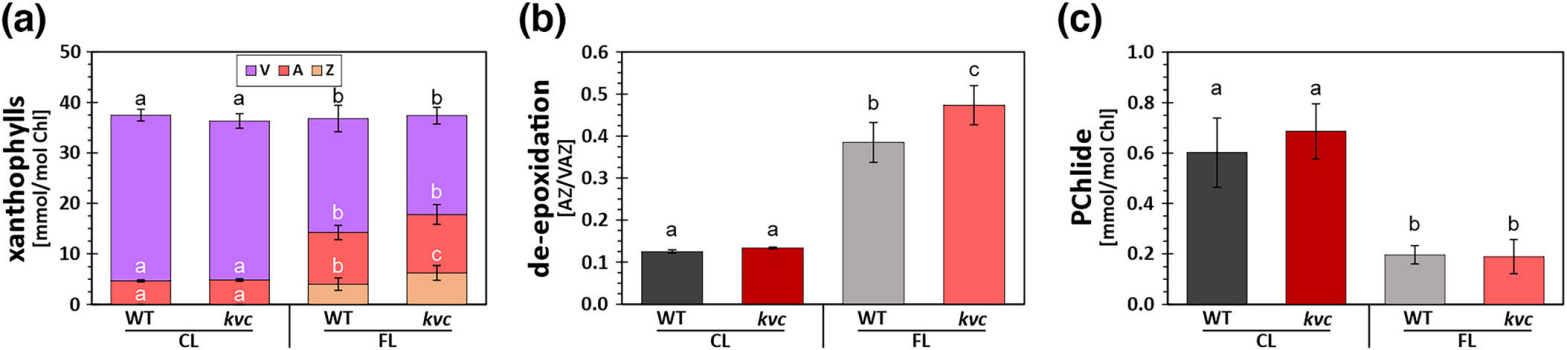
Abundance of xanthophylls and protochlorophyllide. (a) Content of violaxanthin (V), antheraxanthin (A), and zeaxanthin (Z) xanthophylls in WT and *kvc* leaves treated with 6 h constant light (CL) or fluctuating light (FL). (b) Abundance of quenching xanthophylls (Z + A) relative to total xanthophyll content (Z + A + V) in WT and *kvc* leaves treated with 6 h CL or 6 h FL. (c) Abundance of protochlorophyllide (Pchlide) in WT and *kvc* leaves treated with 6 h CL or 6 h FL. Data represent mean with letters indicating statistically significant groups (ANOVA, Tukey‐HSD, *P* < 0.05, error bars denote SD, *n* = 5).

To further investigate the mechanism behind the observed effects of 6 h FL treatment on NPQ, the carotenoids and chlorophylls in mature WT and *kvc* mutant leaves were measured. The overall profile of xanthophyll pigments was clearly changed by FL exposure as compared to CL, with strong decrease of V and increases in the levels of antheraxanthin (A) and Z in both WT and *kvc* (Figure 4a). This resulted in a threefold increase in the de‐epoxidation state between CL and FL in both genotypes (Figure 4b) with *kvc* showing a significantly higher ratio compared with WT. This analysis also demonstrated a small but significantly higher Z content in *kvc* than in WT, whereas total xanthophyll levels (V + A + Z) did not change after FL treatment (Figure 4a). The abundance of protochlorophyllide (Pchlide), a chlorophyll precursor, was substantially diminished after 6 h FL treatment as compared with CL conditions (Figure 4c), although the chlorophyll content and *a/b* ratios were equivalent between the genotypes (Figure S9A,B).

### 3.5 FL causes a decrease in starch contents of leaves

**FIGURE 5:**
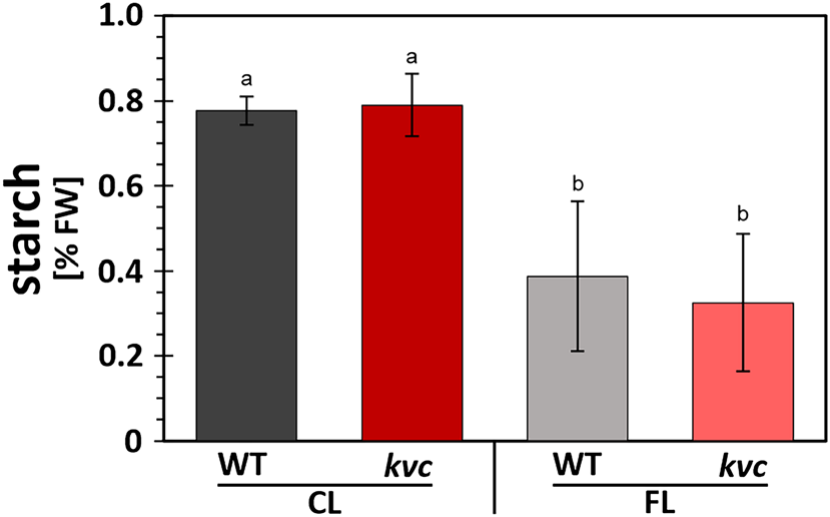
Starch accumulation in leaves after CL and FL treatments. Abundance of starch in WT and *kvc* leaves after 6 h exposure to either constant light (CL) or fluctuating light (FL) conditions. Data represent mean with letters indicating statistically significant groups (ANOVA, Tukey‐HSD, P < 0.05, error bars denote SD, *n* = 3).

The observed effects of FL on photosynthesis prompted us to investigate starch accumulation after 6 h FL exposure in WT and *kvc* leaves. Relative starch content per fresh weight after FL exposure was approximately 50% lower in WT and *kvc*, whereas there was no difference between the two genotypes under either condition (Figure 5).

### 3.6 Cuticle analysis

**FIGURE 6:**
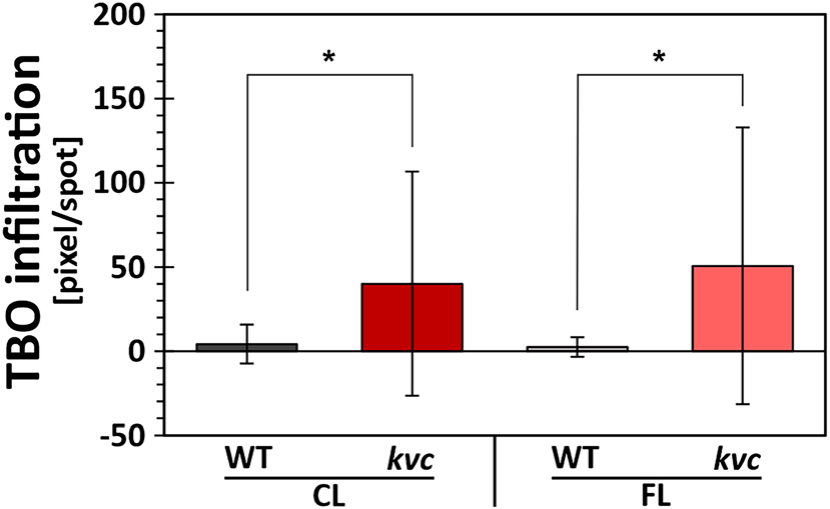
Cuticle permeability in leaves after CL and FL treatments. Quantification of the pixels stained with toluidine blue (TBO) in leaves of WT and *kvc* treated with either 6 h constant light (CL) or 6 h fluctuating light (FL). Data represent mean with (*) indicating statistically significant differences (two‐sample unequal variance t‐test, *P* < 0.05, error bars denote SD, *n* = 16).

The observed upregulation by FL treatment of genes involved in wax and fatty acid synthesis pathways (Table 2) prompted an investigation of the integrity of the leaf cuticle. A leaf drop assay using toluidine blue (TBO) demonstrated significantly higher cuticle permeability in the *kvc* mutant treated with either CL or FL in comparison with WT leaves (Figure 6).

## 4 DISCUSSION

### 4.1 Short‐term FL treatment enhances photoprotective dissipation of excitation energy in both photosystems

This study focused on the dynamic photosynthetic responses to fluctuations in photon flux density. The comparison of WT and *kvc* exposed for 6 h to either CL or FL treatment revealed common rapid and reversible changes in the energy distribution of PSII and PSI during a single simulated FL regime. During the LL‐HL transition, these comprised in both WT and *kvc* of a rapid decrease in Y_II_ (Figure 2a) and Y_I_ (Figure 2d) and concomitant increase in Y_NO_ (Figure 2b) and Y_NA_ (Figure 2e), both of which were subsequently replaced by increasing Y_NPQ_ (Figure 2c) and Y_ND_ (Figure 2f) for the remainder of the HL phase. Although subsequent replacements were already present in CL‐treated plants, they became more pronounced after the FL treatment in both WT and *kvc*. These results, together with similar responses of the single mutants *kea3*, *vccn1*, and clce (Figure S5–S6), underlined that plants actively enhance the photoprotective energy dissipation of both photosystems upon the FL treatment and in turn decrease the energy dissipation via pathways associated with photoinhibition. Y_NO_ comprises the rate constants of fluorescence and unregulated basal dissipation processes within PSII (Hendrickson et al., 2004; Kramer et al., 2004), the latter also including the formation of chlorophyll triplet states (^3^Chl) and singlet oxygen (^1^O_2_) leading to PSII photoinhibition. For PSI, the dissipation via pathways associated with photoinhibition is Y_NA_, which estimates the reduced P700 fraction or PSI acceptor‐side limitation (Klughammer & Schreiber, 2008), leading to ROS production and causing oxidative damage at the iron–sulfur clusters within PSI (Tiwari et al., 2016).

The downregulation of the quantum yields associated with oxidative damage is driven by the progressive upregulation of photoprotective dissipation pathways in both photosystems, which are triggered concomitantly with an increase of lumen acidification (ΔpH) during the HL phase. The increase in ΔpH leads to activation of energy‐dependent NPQ mechanisms (qE) enhanced by both PSBS and Z (Sacharz et al., 2017) causing the subsequent increase in Y_NPQ_. In parallel, the increase in ΔpH also leads to increased photosynthetic control, resulting in a slow‐down of linear electron transfer at the Cytbf complex (Tikhonov, 2014). This caused donor‐side limitation of PSI and accumulation of oxidized PSI (P700^+^), resulting in the subsequent increase in Y_ND_ and harmless dissipation of excess energy as heat by P700^+^ (Schlodder et al., 2005). It is noteworthy that the regulation of energy distribution in both photosystems during the short HL phase (1 min) of the FL‐regime did not lead to adjustments of respective effective quantum yields of photochemistry (Y_II_, Figure 2a; Y_I_, Figure 2d), suggesting that the immediate regulation response of energy distribution focuses directly on minimization of photodamage rather than adjusting linear electron transport, typically observed under longer FL regimes (Ikeuchi et al., 2014).In the *kvc* mutant, regulation of photoprotective energy dissipation of both photosystems was clearly impeded in comparison with WT. This is in line with previous results showing that *kvc* has an altered partitioning of the PMF into ΔpH and ΔΨ (Dukic et al., 2019; Li et al., 2021). In *kvc*, this led to lower Y_NPQ_ (Figure 2c), due to lower induction of NPQ (Figure S4A), and lower Y_ND_ during HL (Figure 2F) as well as slower NPQ relaxation during the HL‐LL transition compared with WT after both CL and FL treatments (Figures 2c and S4A). These differential features in *kvc* are ascribed to the lack of VCCN1 and consequent loss of Cl^−^ import activity into the lumen, leading to lower ΔpH during the HL phase (Duan et al., 2016; Herdean et al., 2016), as well as to the lack of KEA3 and concomitant loss of K^+^/H^+^ antiport activity leading to increased ΔpH during the HL‐LL transition (Armbruster et al., 2014), which matched the corresponding responses observed in *vccn1* and *kea3* single mutants (Figures S5–S6).

### 4.2 The ion channel/transporter mutant *kvc* is sensitized to PSI photoinhibition during short‐term FL treatment

Impaired regulation of the photoprotective energy dissipation of both photosystems in *kvc* led to reduction of maximal PSII and PSI activity after 6 h FL treatment, marked by a 2% decrease of *F*_*v*_/*F*_*m*_ (Figure 1a) and a 27% decrease of Δ*P*_*m*_ (Figure 1b) as compared with WT. This suggests that the decreased NPQ response of *kvc* was not enough to overwhelm the constantly active PSII repair cycle (Chow & Aro, 2005), therefore accumulating only negligible amount of PSII photoinhibition. Because PSI lacks an efficient repair cycle, the accumulation of PSI photoinhibition is expected and routinely observed under FL conditions also in WT (Kono et al., 2014; Suorsa et al., 2012; Wei et al., 2021). However, the extent of PSI photoinhibition strongly depends on the duration of the FL treatment as well as on the applied interval frequency and used high and low light intensities (Kono & Terashima, 2014; Sejima et al., 2014; Tan et al., 2021; Tikkanen & Grebe, 2018), complicating any direct comparisons on PSI photoinhibition and associated thylakoid protein reorganizations between experiments using different FL treatments. Nevertheless, the 6 h FL treatment employed in this study was comparably short and expected to not induce major changes at thylakoid protein level. For this reason, the WT and *kvc* were grown for 6 weeks under the same FL treatment, followed by an assessment of photosynthetic protein contents. These showed lower PSI content in *kvc* (Figure S7), which further underscored the susceptibility of PSI in *kvc* to PSI photoinhibition in comparison with WT, a phenomenon not observed under constant growth light conditions (Dukic et al., 2022).

It should be noted that in addition to *kvc*, also *clce*, *vccn1*, and *kea3* single mutants showed mild PSI photoinhibition phenotypes after 6 h FL treatment (Figure S2B), suggesting that altered thylakoid ion‐homeostasis might generally affect PSI susceptibility to photoinhibition, with a potential additive effect in the absence of different ion channels/transporters. Nevertheless, based on extensive research on the diverse functions of thylakoid ion channels/transporters regulating photosynthesis (Armbruster et al., 2017; Spetea et al., 2017), the interaction of VCCN1 with CLCE and KEA3 likely explains the slightly enhanced FL‐induced photoinhibition of PSI in *kvc* compared with individual single mutants. Specifically, during the LL‐HL transition, the absence of VCCN1 (Duan et al., 2016; Herdean et al., 2016) would lead to a lower ΔpH and diminished photosynthetic control, which in turn results in more electrons reaching already acceptor‐side limited PSI, hence causing ROS production and PSI photoinhibition during the HL phase (Huang et al., 2019; Tikkanen & Grebe, 2018). It is important to note that PSI photoinhibition is not per‐se detrimental to photosynthesis, because only a drastic decrease in the active PSI content would limit linear electron transport (Schöttler & Tóth, 2014), because of inherent fast forward electron transport reactions within PSI (Brettel & Leibl, 2001). Furthermore, PSI inhibition is mainly caused by an overreduction of the PSI acceptor side, effectively reducing the maximal PSI activity (Sonoike, 2011), which in turn decreases PSI acceptor‐side limitation by increasing the availability of acceptors per PSI. This self‐regulated adjustment likely explains the observed decrease in Y_NA_ (Figure 2e) and increase in Y_ND_ (Figure 2f) in PSI photoinhibited *kvc* after FL treatment similar to WT at the end of the HL phase. Consequently, it can be argued that the moderate extent of PSI photoinhibition in *kvc* mutant did not prevent acclimation to short‐term FL conditions, in line with previous observations (Dukic et al., 2019).

### 4.3 Transcript abundance of photoprotection genes and changes in primary metabolism upon short‐term FL treatment

In both WT and *kvc*, the NPQ response was strongly enhanced by 6 h FL treatment although to a different extent (Figure S4A). Simultaneously, transcript analysis of WT revealed moderate upregulation of the PSBS gene (*NPQ4*, log2 FC = 0.8–1.1) as well as *NPQ1* and *NPQ2* genes (*NPQ1*, FC = log2 0.7–0.9; *NPQ2*, FC = log2 0.6–0.7), expressing the VDE and ZE enzymes responsible for de‐epoxidation and epoxidation of xanthophylls, respectively, and similar transcript abundances were recorded in *kvc* after the FL treatment (Data S1). It is unlikely that changes in protein contents would be directly responsible for rapid changes in the NPQ response upon the short FL treatment. More likely, rapid NPQ changes might be caused by a transient decrease in lumen pH during the HL phase of the FL treatment, also explaining the observed higher accumulation of de‐epoxidized xanthophylls after FL treatment in both WT and *kvc* (Figure 4a,b) (Alter et al., 2012; Wei et al., 2021). The higher accumulation of Z in *kvc* compared with WT is likely linked to the specific loss of KEA3 (von Bismarck et al., 2023). Accumulation of transcripts in both genotypes for β‐hydroxylase (CHY1; Table 2), which converts β‐carotene directly to Z (Rissler & Pogson, 2001), may have also contributed to observed increases in Z after the FL treatment (Figure 4a). Furthermore, enhanced transcript accumulation of several enzymes involved in terpene biosynthesis in both genotypes (Table 2) may support the production of xanthophylls and other terpenoids required for photoprotection during FL‐induced light stress. The slightly higher accumulation of Z after FL treatment in *kvc* might have contributed to increase in NPQ during HL phase (Figures 2c and S4A) but might also be related to its high antioxidant capacity in the thylakoid membrane (Havaux, & Dall'Osto, L., and Bassi, R., 2007), counteracting increased ROS production caused by higher ΔΨ in the first seconds of the LL‐HL transition (Johnson & Ruban, 2014), which normally activates VCCN1 and leads to re‐partitioning of PMF toward ΔpH (Herdean et al., 2016).

Light stress, like other abiotic stress factors, is known to impact starch metabolism (Ribeiro et al., 2022) and could explain the diminished starch accumulation after 6 h FL treatment (Figure 5). Lower starch abundance also relates to enhanced stomatal closure during FL, which decreases CO_2_ fixation and biomass accumulation (Matthews et al., 2018). In the current work, accumulation in 6 h FL‐treated WT and *kvc* of gene transcripts for several enzymes involved in the metabolism of 2‐phosphoglycolate (Table 2), the toxic metabolite produced during photorespiration (Busch, 2020), supports the concept that FL exposure stimulates photorespiration at the expense of CO_2_ fixation due to stomatal closure. Decreased carbon assimilation during FL reduces the consumption of ATP, leading to an increase in the stromal ATP/ADP ratio and a decrease in thylakoid ATP synthase activity (Wei et al., 2021). This is in line with a clear decrease in abundance of ATP synthase subunit AtpF upon growth of Arabidopsis *kvc* mutant under FL conditions (Figure S7) and possibly also contributes to lumen acidification. Decrease in ATP synthase, together with increase in PSBS and VDE abundance (Figure S7), not observed in *kvc* under CL conditions (Li et al., 2021), might be part of the long‐term FL acclimation responses of *kvc*. Lower ATP synthase contents have been generally associated with increase in PMF (Kanazawa et al., 2017; Rott et al., 2011), which in the case of *kvc* might compensate for the lack of PMF modulation via ion channels/transporters and in turn increase the ΔpH‐dependent NPQ response after long‐term FL acclimation.

### 4.4 Signaling and stress responses upon short‐term FL treatment

The major response at transcript level to the 6 h FL treatment, in WT and *kvc*, comprised the upregulation of a number of genes involved in diverse secondary metabolism pathways (Tables 1, 2), which were largely similar in *clce*, *kea3*, and *vccn1* single mutants (Tables S1–S2). This prompted us to focus the investigation on only WT and *kvc*. The simultaneous induction of herbivory and biotic stress response pathways in WT and *kvc* (Table 1), as well as specific components of JA signaling (Table 2), suggest the activity of oxylipin signaling under FL. Furthermore, the signaling effects of 12‐oxophytodienoic acid (OPDA), a JA precursor, could likewise account for the upregulation of abiotic stress‐related genes detailed in Table 2, being often ascribed to H_2_O_2_ signaling (Gollan & Aro, 2020), but can also be activated by ^1^O_2_ (Ramel et al., 2012). Increased ^1^O_2_ production is also supported by upregulation of tocopherol synthesis genes during FL in both genotypes (Table 2). The main source of ^1^O_2_ in chloroplasts is over‐excitation of PSII leading to ^3^Chl formation (Khorobrykh et al., 2020), which is supported by accumulation of reduced (closed) PSII centers during the HL phase in both genotypes (Figure S4B).

Nevertheless, the apparently higher ^1^O_2_ levels during 6‐h FL treatment, in comparison with CL, caused only a minor decrease in *F*_*v*_/*F*_*m*_ (Figure 1a), implying that the rate of constitutive PSII repair kept pace with PSII photoinhibition. Albeit no change in total chlorophyll content (Figure S9A) was observed, lower Pchlide levels (Figure 4c) and lower expression of Pchlide reductase (POR) after FL treatment (Data S1) predict a reduced synthesis of 5‐aminolevulinic acid, the rate‐limiting step for chlorophyll synthesis (Wang et al., 2022), which might prevent accumulation of photoreactive tetrapyrrole metabolites, otherwise providing an opportunity for ^1^O_2_ production during the turnover of photosensitive chlorophyll (Wagner et al., 2004). Taking these results together, it is conceivable that, despite enhanced NPQ induction by FL, PSII over‐excitation and subsequent ^1^O_2_ signaling are likely major contributors to the FL regulation in both WT and *kvc*. Additionally, the mild PSI photoinhibition in *kvc* did not result in a specific transcriptome response compared to WT. Previously reported upregulation of chloroplastic iron chaperones FER1, FER3, and FER4 in PSI photoinhibited *pgr5* mutant (Gollan et al., 2017) were found to be significantly upregulated (log2 FC = 1.2–1.7) in both WT and *kvc* after FL treatment (Data S1). This might be explained by relatively early photoinhibition of PSI during the FL treatment leading to rapid self‐adjustment of energy distribution within PSI (see above) similar to WT (Figure 2d–f), which terminated PSI‐dependent ROS signaling and a transcriptomic response to PSI photoinhibition (Lima‐Melo et al., 2021).

Surprisingly, only a limited set of genes was DE in the *kvc* mutant under CL as compared with WT (Data S1). Of these, the upregulated genes in *kvc* versus WT comparison showed a substantial correlation with genes upregulated by FL in both genotypes (Figure 3d). This finding was even more striking when analyzing the GO terms induced by the *kvc* mutation in CL, which described biotic stress, defense responses, and JA signaling (Data S2) and were found to overlap almost completely with GO terms induced by FL (Figure 3e). This implies that biotic stress‐responsive signaling pathways induced by FL in WT were already partially active in *kvc* under CL (non‐stress) conditions. Similar upregulation of GO‐terms in *clce* and *vccn1*, but not *kea3*, already under CL conditions (Table S1) suggests that biotic stress‐responsive signaling pathways might be linked to altered thylakoid Cl^−^ ion‐homeostasis, which should be further investigated.In *kvc*, this observation may also relate to the results of cuticle integrity analysis, which, despite upregulation of genes involved in synthesis of long‐chain fatty acids (LCFAs), wax, and cutin in both genotypes (Table 2), did not reveal any changes in permeability after the FL treatment (Figure 6), yet a higher cuticle permeability was evident in *kvc* in comparison with WT after both CL and FL treatments. Therefore, the upregulated transcription of cuticle fortification genes under FL may relate to other FL‐induced processes, such as decreased CO_2_ fixation, altered stomatal conductance, or upregulation of biotic stress response. It is also possible that changes in cuticle permeability may appear at a later time point that was not investigated here.

The leaf cuticle is implicated in defense against biotic infection, both by presenting a physical barrier to microbes and through regulation of host defense signaling. Compromised cuticle integrity correlates with increased resistance against the fungal pathogen *Botrytis cinerea*, due to enhanced generation of ROS and primed sensitivity to pathogenesis (Cui et al., 2019; L'Haridon et al., 2011). Cuticle permeability detected in *kvc* in the current study, alongside upregulation of genetic pathways associated with biotic stress (Tables 1 and 2), suggests that pathogen resistance may be induced, to some degree, in *kvc*. In light of these observations, including high expression of JA regulators ILL5 and JOX2 in *kvc* under CL (Table 2), it is tempting to speculate that oxylipin signaling was active in *kvc* under CL conditions, although this calls for further investigation, also in single ion‐channel/transporter mutants.

## 5 CONCLUSIONS

Reported dynamic changes in energy distribution of PSII and PSI illustrate that even short exposure of Arabidopsis to FL conditions enhances photoprotective dissipation of excitation energy in both photosystems linked to thylakoid ion transport and lumen acidification. These findings are in line with enhancement of PMF partitioning to ΔpH by FL (Wei et al., 2021), yet the current study clearly demonstrates that these effects are activated already within a few hours of FL exposure. Acclimation responses in FL‐exposed plants are likely involved in protecting PSII and PSI from photodamage induced by continuously alternating HL and LL phases. Because of the clear role of thylakoid H^+^ gradient in FL response, we also investigated the acclimation of the *kvc* mutant (Dukic et al., 2019), which lacks both Cl^−^ influx during LL‐HL transition (VCCN1) and H^+^ efflux during HL‐LL transition (KEA3). In addition to the previously described effects on NPQ induction and relaxation, FL exposure of *kvc* induced a moderate extent of PSI photoinhibition, which is suggested to lead to self‐regulated adjustments of PSI excitation energy distribution.FL treatment upregulated biotic stress response and secondary metabolism, including cuticle and terpenoid biosynthesis, which appear to involve ^1^O_2_ signaling pathway traced to PSII over‐reduction that occurs during FL. Notably, neither PSII photoinhibition, chlorophyll turnover nor gene expression was substantially different between WT and *kvc* plants exposed to FL, which reiterates the robustness of acclimation to unstable conditions found in nature. However, upregulation of FL‐induced genes in *kvc* mutant under CL conditions suggests a so far unexplored indirect link between thylakoid ion channels/transporters and biotic stress response signaling that remains to be investigated.

## AUTHOR CONTRIBUTIONS

Peter J. Gollan, Steffen Grebe, Cornelia Spetea, and Eva‐Mari Aro designed research. Peter J. Gollan, Steffen Grebe, and Lena Roling performed research. Peter J. Gollan, Steffen Grebe, Lena Roling, Bernhard Grimm, and Eva‐Mari Aro analyzed data. Peter J. Gollan, Steffen Grebe, and Eva‐Mari Aro wrote the manuscript. All authors reviewed and edited the manuscript.

## CONFLICT OF INTEREST STATEMENT

The Authors did not report any conflict of interest.

## PEER REVIEW

The peer review history for this article is available in the Supporting Information for this article.

## Supporting information


**Data S1.** Supporting Information.

**Data S2.** Supporting Information.

**Table S1.** Gene Ontology (GO) terms significantly enriched in up‐ or down‐regulated genes of WT, *kvc* and single mutants *kea3*, *vccn1*, *clce*.
**Table S2.** Genes demonstrating significantly differential expression between WT, *kvc* and single mutants *kea3, vccn1 and clce* as induced by exposing the plants to FL conditions.
**Figure S1.** Validation of far‐red method for F_**0**_ **acquisition.**
**Figure S2.** Maximal PSII quantum yield and maximal redox active PSI fraction of WT, *kvc* triple mutant and *clce*, *kea3*, *vccn1* single mutants after CL and FL treatment.
**Figure S3.** Fluo and P700 traces of WT and *kvc* after CL and FL treatment.
**Figure S4.** Changes of NPQ and qL in WT and *kvc* after CL and FL treatment.
**Figure S5.** Changes in PSII and PSI quantum yields in WT, *kvc* triple mutant and *clce*, *kea3* and *vccn1* single mutants after CL treatment.
**Figure S6.** Changes in PSII and PSI quantum yields in WT, *kvc* triple mutant and *clce*, *kea3* and *vccn1* single mutants after FL treatment.
**Figure S7.** Changes of thylakoid protein abundance in WT and *kvc* grown in FL conditions.
**Figure S8.** Common and distinct expression profiles induced by fluctuating light and other stresses.
**Figure S9.** Total chlorophyll content and chlorophyll a/b ratio.

**Data S4.** Supporting Information.

**Data S5.** Peer Review.

## Data Availability

The RNA‐seq data for Arabidopsis WT and *kvc* are available at the NCBI under project PRJNA735049.
