# Supplementary material for: Photosynthetic and transcriptome responses to fluctuating light in Arabidopsis thylakoid ion transport triple mutant
Source: Plant Direct. 2023 Oct 25;7(10):e534. doi: 10.1002/pld3.534 (PMC10598627; doi:10.1002/pld3.534)
Supplement: Supplementary file 3 — Table S1. Gene Ontology (GO) terms significantly enriched in up‐ or down‐regulated genes of WT, kvc and single mutants kea3, vccn1, clce. Table S2. Genes demonstrating significantly differential expression between WT, kvc and single mutants kea3, vccn1 and clce as induced by exposing the plants to FL conditions. Figure S1. Validation of far‐red method for F 0 acquisition. Figure S2. Maximal PSII quantum yield and maximal redox active PSI fraction of WT, kvc triple mutant and clce, kea3, vccn1 single mutants after CL and FL treatment. Figure S3. Fluo and P700 traces of WT and kvc after CL and FL treatment. Figure S4. Changes of NPQ and qL in WT and kvc after CL and FL treatment. Figure S5. Changes in PSII and PSI quantum yields in WT, kvc triple mutant and clce, kea3 and vccn1 single mutants after CL treatment. Figure S6. Changes in PSII and PSI quantum yields in WT, kvc triple mutant and clce, kea3 and vccn1 single mutants after FL treatment. Figure S7. Changes of thylakoid protein abundance in WT and kvc grown in FL conditions. Figure S8. Common and distinct expression profiles induced by fluctuating light and other stresses. Figure S9. Total chlorophyll content and chlorophyll a/b ratio. [file PLD3-7-e534-s001.docx]

Supplemental materials for

**Photosynthetic and transcriptome responses to fluctuating light in Arabidopsis thylakoid ion transport triple mutant**

Peter J. Gollan^1^, Steffen Grebe*^,1,4^, Lena Roling^2^, Bernhard Grimm^2^, Cornelia Spetea^3^, Eva-Mari Aro*^1^

^1^ Department of Life Technologies, Molecular Plant Biology, University of Turku, Turku, Finland

^2^ Institute of Biology/Plant Physiology, Humboldt-Universität zu Berlin, Berlin, Germany

^3^ Department of Biological and Environmental Sciences, University of Gothenburg, Gothenburg, Sweden

^4^ Present address: Optics of Photosynthesis Laboratory, Institute for Atmospheric and Earth System Research (INAR)/Forest Sciences, Viikki Plant Science Center (ViPS), University of Helsinki, Helsinki, Finland

* Correspondence: steffen.grebe@helsinki.fi; evaaro@utu.fi

**This document contains:**

Supplemental Table S1
Supplemental Table S2

Supplemental Figures S1-S9

Legends for Supplemental data Files 1 and 2

**Other supplemental materials for this manuscript include the following:**

Supplemental data Files 1 and 2

**Supplemental Table S1**. Gene Ontology (GO) terms significantly enriched in up- or down-regulated genes of WT, *kvc* and single mutants *kea3*, *vccn1*, *clce*.

| **GO term** | **GO term ID** | **Fold enrichment WT FL vs CL** | **Fold enrichment *kvc* FL vs CL** | **Fold enrichment *kea3* FL vs CL** | **Fold enrichment *vccn1* FL vs CL** | **Fold enrichment *clce* FL vs CL** | **Fold enrichment *kvc* vs WT CL** | **Fold enrichment *kea3* vs WT CL** | **Fold enrichment *vccn1* vs WT CL** | **Fold enrichment *clce* vs WT CL** |
| --- | --- | --- | --- | --- | --- | --- | --- | --- | --- | --- |
| **Wax biosynthesis** | GO:0010025 | 7.8 | 6.1 | 5.1 | 4.3 | 4.9 | n.s. | n.s. | n.s. | n.s. |
| **Response to hypoxia** | GO:0001666 | 6.2 | 5.0 | 5.1 | 4.0 | 4.5 | n.s. | n.s. | 4.0 | n.s. |
| **Terpenoid metabolism** | GO:0006721 | 3.3 | 3.6 | 2.4 | 2.4 | 2.8 | n.s. | n.s. | n.s. | n.s. |
| **Response to herbivore** | GO:0080027 | 10.9 | 8.0 | 8.5 | 6.8 | 6.2 | n.s. | n.s. | n.s. | n.s. |
| **Response to biotic stimulus** | GO:0009607 | 3.8 | 3.3 | 2.9 | 2.4 | 2.8 | 7.3 | n.s. | 2.6 | 3.4 |
| **Defense response** | GO:0006952 | 3.3 | 3.1 | 2.9 | 2.3 | 2.6 | 6.7 | n.s. | 2.8 | 3.2 |
| **DNA  replication** | GO:0006260 | 9.5 | 5.0 | n.s. | n.s. | n.s. | n.s. | n.s. | n.s. | n.s. |
| **Regulation of SA signalling** | GO:2000031 | 8.9 | 9.0 | n.s. | 5.3 | n.s. | n.s. | n.s. | n.s. | n.s. |
| **SA mediated signalling pathway** | GO:0009863 | 3.6 | 6.0 | 3.8 | 3.4 | 3.3 | n.s. | n.s. | n.s. | n.s. |

GO terms enriched in upregulated genes coloured light blue, GO terms enriched in down-regulated genes coloured dark blue, n.s. not significant.

**Supplemental Table S2**. Genes demonstrating significantly differential expression between WT, *kvc* and single mutants *kea3, vccn1 and clce* as induced by exposing the plants to FL conditions.

| **Name** | **AGI code** | **Description** | **log2FC**  **FL vs CL WT** | **log2FC**  **FL vs CL kvc** | **log2FC**  **FL vs CL *kea3*** | **log2FC**  **FL vs CL *vccn1*** | **log2FC**  **FL vs CL *clce*** |
| --- | --- | --- | --- | --- | --- | --- | --- |
| ***Terpenoid synthesis*** | | | | | | | |
| GGPPS7 | AT2G18620 | Terpene biosynthesis | 2.3 | 1.7 | 2.7 | 2.4 | 2.2 |
| SPS1 | AT1G78510 |  | 1.2 | 1.2 | 1.4 | 1.2 | 1.3 |
| SPS2 | AT1G17050 |  | 1.1 | 1.3 | 1.3 | 1.6 | 1.4 |
| TPS03 | AT4G16740 |  | 3.3 | 2.4 | 2.9 | 3.3 | 2.9 |
| TPS10 | AT2G24210 |  | 2.8 | 1.9 | 3.0 | 1.6 | 3.3 |
| GES | AT1G61120 |  | 3.9 | 1.0 | 3.0 | 1.2 | 0.7 |
| CHY1 | AT4G25700 | Xanthophyll synthesis from  β-carotene | 1.7 | 2.2 | 2.1 | 2.3 | 2.2 |
| VTE1 | AT4G32770 | Tocopherol/quinone synthesis | 1.3 | 1.2 | 1.4 | 1.3 | 1.2 |
| PDS1 | AT1G06570 |  | 1.3 | 0.9 | 0.9 | 0.6 | 0.7 |
| CPT4/CTP7 | AT5G58770 | Polyprenol synthesis | 1.1 | 0.7 | 1.3 | 1.2 | 1.2 |
| ***Fatty acid, wax synthesis*** | | | | | | | |
| KCS1 | AT1G01120 | Long chain fatty acid synthesis /elongation | 1.5 | 1.3 | 1.4 | 1.3 | 1.5 |
| KCS2 | AT1G04220 |  | 2.9 | 2.1 | 2.4 | 2.2 | 1.9 |
| KCS3 | AT1G07720 |  | 1.3 | 1.0 | 1.2 | 1.1 | 1.2 |
| KCS5 | AT1G25450 |  | 1.3 | 1.1 | 1.5 | 1.4 | 1.1 |
| KCS6 | AT1G68530 |  | 1.4 | 0.8 | 0.9 | 0.7 | 0.7 |
| KCS8 | AT2G15090 |  | 2.8 | 2.5 | 1.9 | 2.0 | 2.3 |
| KCS12 | AT2G28630 |  | 3.2 | 3.3 | 2.4 | 3.3 | 2.9 |
| KCS19 | AT5G04530 |  | -2.8 | -2.3 | -1.8 | -2.1 | -2.4 |
| KCS20 | AT5G43760 |  | 1.1 | 0.5 | 0.7 | 0.5 | 0.7 |
| KCR2 | AT1G24470 |  | 4.4 | 3.7 | 4.1 | 3.5 | 3.4 |
| FABG | AT3G04000 |  | 1.6 | 1.6 | 0.8 | 1.3 | 0.5 |
| CER8 (LACS1) | AT2G47240 |  | 2.6 | 3.4 | 2.9 | 3.1 | 3.0 |
| LACS3 | AT1G64400 |  | 1.9 | 2.0 | 2.0 | 2.1 | 2.0 |
| AT5G47330 | AT5G47330 |  | 2.9 | 3.6 | 3.5 | 3.4 | 3.3 |
| CER1 | AT1G02205 | Wax/suberin/cutin synthesis and secretion | 1.9 | 0.6 | 1.2 | 0.7 | 1.0 |
| CER3 (WAX2) | AT5G57800 |  | 1.1 | 1.2 | 1.2 | 1.4 | 1.4 |
| FAR1 | AT5G22500 |  | 1.7 | 1.1 | 0.5 | 0.3 | 0.1 |
| WSD1 | AT5G37300 |  | 4.4 | 3.3 | 3.7 | 4.5 | 3.6 |
| WDS6 | AT3G49210 |  | 2.1 | 2.0 | 2.1 | 1.9 | 2.0 |
| WSD-like | AT5G16350 |  | -0.9 | -0.2 | n.d. | n.d. | n.d. |
| MAH1 | AT1G57750 |  | 3.1 | 2.4 | 3.0 | 1.9 | 2.6 |
| ***Berberine bridge enzyme (BBE)-like domain-containing protein*** | | | | | | | |
| AtBBE3 | AT1G26380 | Oxidation of secondary metabolites | 5.0 | 5.7 | 5.2 | 5.6 | 5.3 |
| AtBBE6 | AT1G26410 |  | 4.4 | 4.8 | 4.0 | 4.2 | 3.8 |
| AtBBE7 | AT1G26420 |  | 7.2 | 5.5 | 5.2 | 6.2 | 5.7 |
| AtBBE8 | AT1G30700 |  | 3.8 | 3.6 | 4.1 | 3.2 | 3.5 |
| AtBBE9 | AT1G30720 |  | 7.2 | 6.7 | 6.5 | 5.2 | 5.8 |
| AtBBE11 | AT1G30730 |  | 6.2 | 6.0 | 5.3 | 5.5 | 5.0 |
| AtBBE18 | AT4G20860 |  | 3.2 | 3.3 | 3.4 | 3.1 | 3.1 |
| AtBBE22 | AT4G20820 |  | 2.3 | 1.0 | 0.7 | 0.7 | 0.7 |
| AT5G44380 | AT5G44380 |  | 2.2 | 2.0 | 2.4 | 1.4 | 0.9 |
| ***Regulation of hormone signalling*** | | | | | | | |
| NCED3 | AT3G14440 | Abscisic acid synthesis/catabolism | 1.2 | 1.0 | 0.7 | 0.3 | 0.5 |
| NCED5 | AT1G30100 |  | 1.5 | 0.6 | 1.0 | 0.7 | 0.9 |
| CYP707A2 | AT2G29090 |  | 1.8 | 1.6 | 1.6 | 1.5 | 1.3 |
| CYP707A3 | AT5G45340 |  | 4.8 | 1.7 | 4.5 | 3.8 | 4.5 |
| ABA2 | AT1G52340 |  | -1.1 | -0.7 | n.d. | n.d. | n.d. |
| GA2OX1 | AT1G78440 | Gibberellic acid oxygenase | 1.2 | 1.8 | 2.0 | 2.0 | 2.0 |
| GA2OX4 | AT1G02400 |  | 4.8 | 4.6 | 3.9 | 4.1 | 3.3 |
| GA2OX8 | AT4G21200 |  | 0.9 | 2.1 | 2.6 | 2.4 | 1.7 |
| GA3OX1 | AT1G15550 |  | 2.4 | 2.8 | 2.7 | 2.4 | 2.5 |
| GA3OX2 | AT1G80340 |  | 1.7 | 2.4 | 1.9 | 1.7 | 2.3 |
| GA20OX1 | AT4G25420 |  | -1.7 | -1.7 | -1.8 | -2.0 | -1.8 |
| GA20OX2 | AT5G51810 |  | 1.7 | 1.5 | 1.6 | 0.7 | 1.3 |
| JOX2 | AT5G05600 | Jasmonic acid oxygenase | 2.1 | 0.3 | 2.0 | 2.1 | 1.3 |
| JOX3/JRG21 | AT3G55970 |  | 3.6 | 1.6 | 3.5 | 2.6 | 3.2 |
| JOX4 | AT2G38240 |  | 4.2 | 3.7 | 3.3 | 2.7 | 2.6 |
| CYP94B1 | AT5G63450 | Jasmonic acid-Ile turnover | 3.1 | 1.3 | 2.8 | 2.3 | 1.8 |
| CYP94C1 | AT2G27690 |  | 1.5 | 0.6 | 1.5 | 1.6 | 1.4 |
| ILL5 | AT1G51780 |  | 7.2 | 3.9 | 6.6 | 5.0 | 5.6 |
| OPR-like | AT1G18020 | 12-oxophytodienoic acid reduction, jasmonic acid synthesis | -1.9 | -2.3 | -2.3 | -2.4 | -2.6 |
| OPR-like | AT1G09400 |  | -1.3 | -2.5 | -1.8 | -1.9 | -1.4 |
| OPR-like | AT1G17990 |  | -2.2 | -2.1 | -2.2 | -2.4 | -2.6 |
| ***Abiotic stress-related transcription regulators*** | | | | | | | |
| HSFA3 | AT5G03720 | Heat shock transcription factor | 1.7 | 1.9 | 1.9 | 1.7 | 1.8 |
| HSFA6A | AT5G43840 |  | 2.5 | 1.5 | 0.5 | 0.6 | 1.2 |
| HSFA6B | AT3G22830 |  | 1.6 | 0.0 | 1.5 | 0.3 | 0.4 |
| HSFA8 | AT1G67970 |  | 1.4 | 1.0 | 1.1 | 0.7 | 0.9 |
| HSFB2A | AT5G62020 |  | 1.3 | 0.8 | 0.9 | 0.8 | 0.6 |
| DREB2A | AT5G05410 | Dehydration-responsive element-binding protein | 2.0 | 1.7 | 2.1 | 1.4 | 1.7 |
| DREB2B | AT3G11020 |  | 1.2 | 1.1 | 1.1 | 1.0 | 1.1 |
| DREB2C | AT2G40340 |  | 3.1 | 2.2 | 2.7 | 2.6 | 2.3 |
| DREB2E | AT2G38340 |  | 2.6 | 1.6 | n.d. | n.d. | n.d. |
| ZAT10 | AT1G27730 | Zinc finger transcription factor | 4.4 | 4.1 | 3.3 | 2.7 | 2.4 |
| ZAT12 | AT5G59820 |  | 5.1 | 4.1 | 4.8 | 3.6 | 3.6 |
| ABI5 | AT2G36270 | Abscisic acid-responsive transcription | 1.4 | -0.1 | 0.5 | 0.3 | 0.3 |
| NAC046 | AT3G04060 | NAC domain-containing regulator | 2.7 | 0.7 | 1.2 | 0.3 | 1.0 |
| ***Photorespiration*** | | | | | | | |
| GOX1 | AT3G14420 | Glycolate/glyoxylate catabolism | 1.0 | 1.1 | n.d. | n.d. | n.d. |
| AGT1 | AT2G13360 |  | 1.1 | 1.1 | 0.9 | 1.0 | 1.0 |
| AGT3 | AT2G38400 |  | 1.6 | 1.9 | 1.8 | 1.7 | 1.8 |
| GGT2 | AT1G70580 |  | 1.2 | 1.4 | n.d. | n.d. | n.d. |
| GLPD2 | AT2G26080 |  | 1.7 | 1.8 | 1.6 | 1.5 | 1.4 |
| GLN1;1 | AT5G37600 |  | 1.4 | 1.7 | 1.6 | 1.5 | 1.4 |

Gene expression >2FC upregulated coloured light blue, >2FC down-regulated coloured dark blue; n.d. - no data.

**
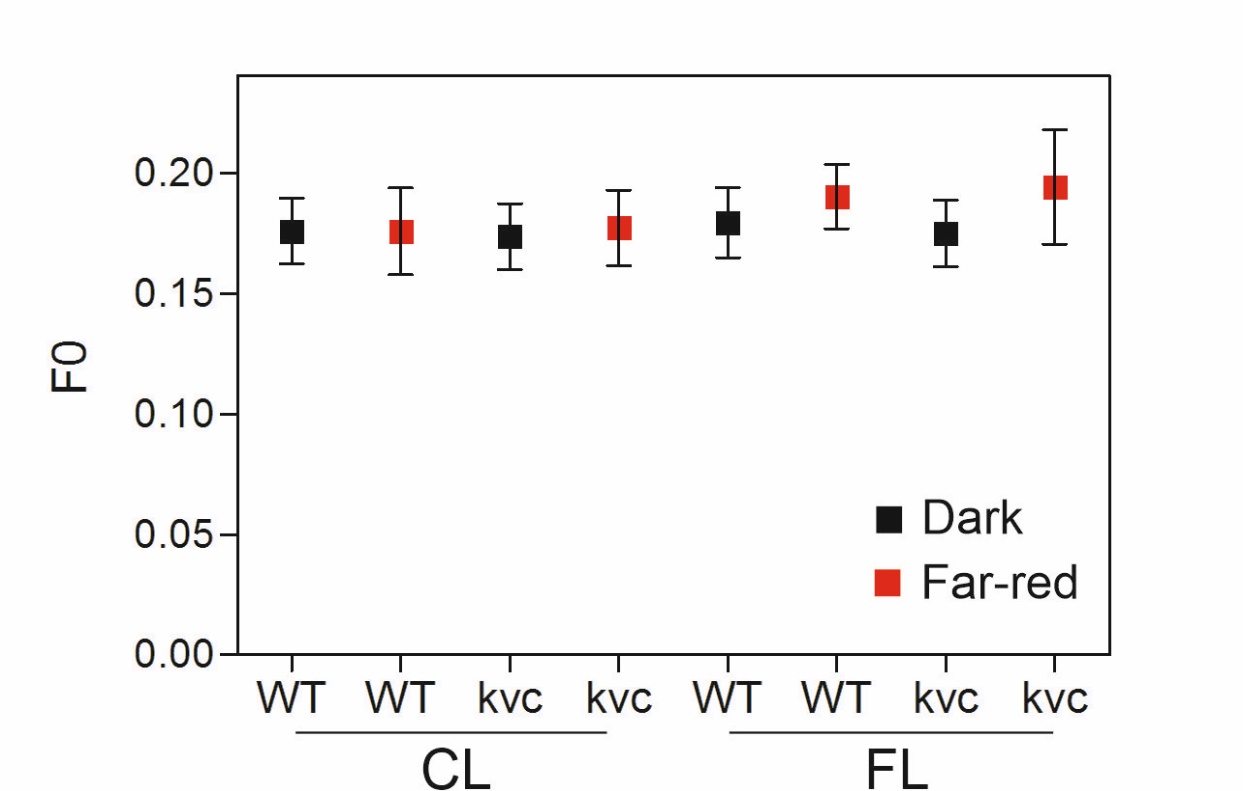
**

**Supplemental Figure S1: Validation of far-red method for F_0_ acquisition.**

Wild type (WT) and *kvc* triple mutant plants were grown for 5 weeks under 8 h/16 h light/dark cycle and a light intensity of 100 µmol photons m^-2^ s^-1^. F_0_ values measured in dark-adapted leaves and after far-red light treatment, as described in Methods. T-test between samples showed no significant differences, error bars indicate standard deviations; n=4.


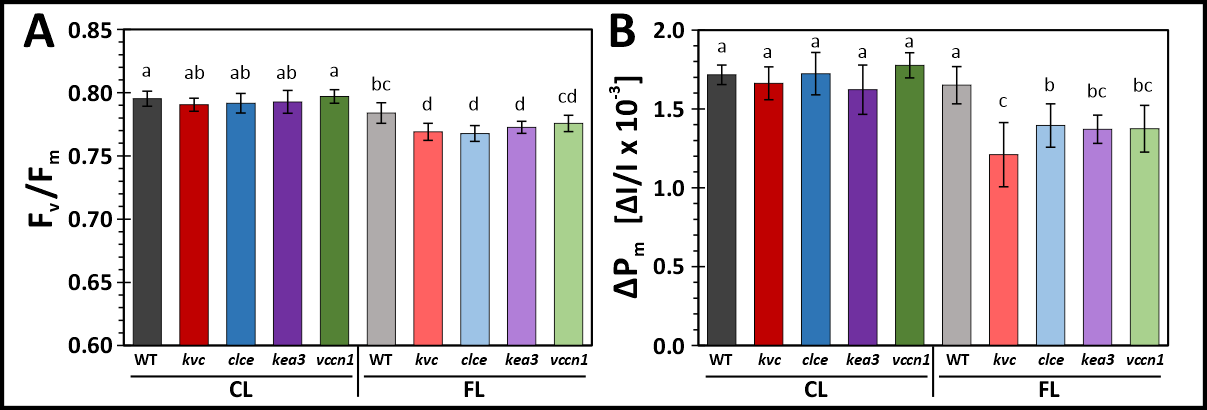


**Supplemental Figure S2: Maximal PSII quantum yield and maximal redox active PSI fraction of WT, *kvc* triple mutant and *clce*, *kea3*, *vccn1* single mutants after CL and FL treatment.**

Estimation of (A) maximal quantum yield of photochemistry in PSII (F_v_/F_m_) and (B) maximal redox active fraction of PSI (ΔP_m_) of dark acclimated wild type (WT, grey), *kvc* triple mutant (*kvc*, red), *clce* single mutant (*clce*, blue), *kea3* single mutant (*kea3*, purple) and *vccn1* single mutant (*vccn1*, green) after treatment for 6 h with constant light (CL, 100 µmol photons m-2 s-1) or 6h with fluctuating light (FL, 50 µmol photons m-2 s-1 for 4 min and 500 µmol photons m-2 s-1 for 1 min). Data represents mean with letters indicating statistically significant groups (ANOVA, Tukey-HSD, P<0.05, error bars denote SD, n=10-12).


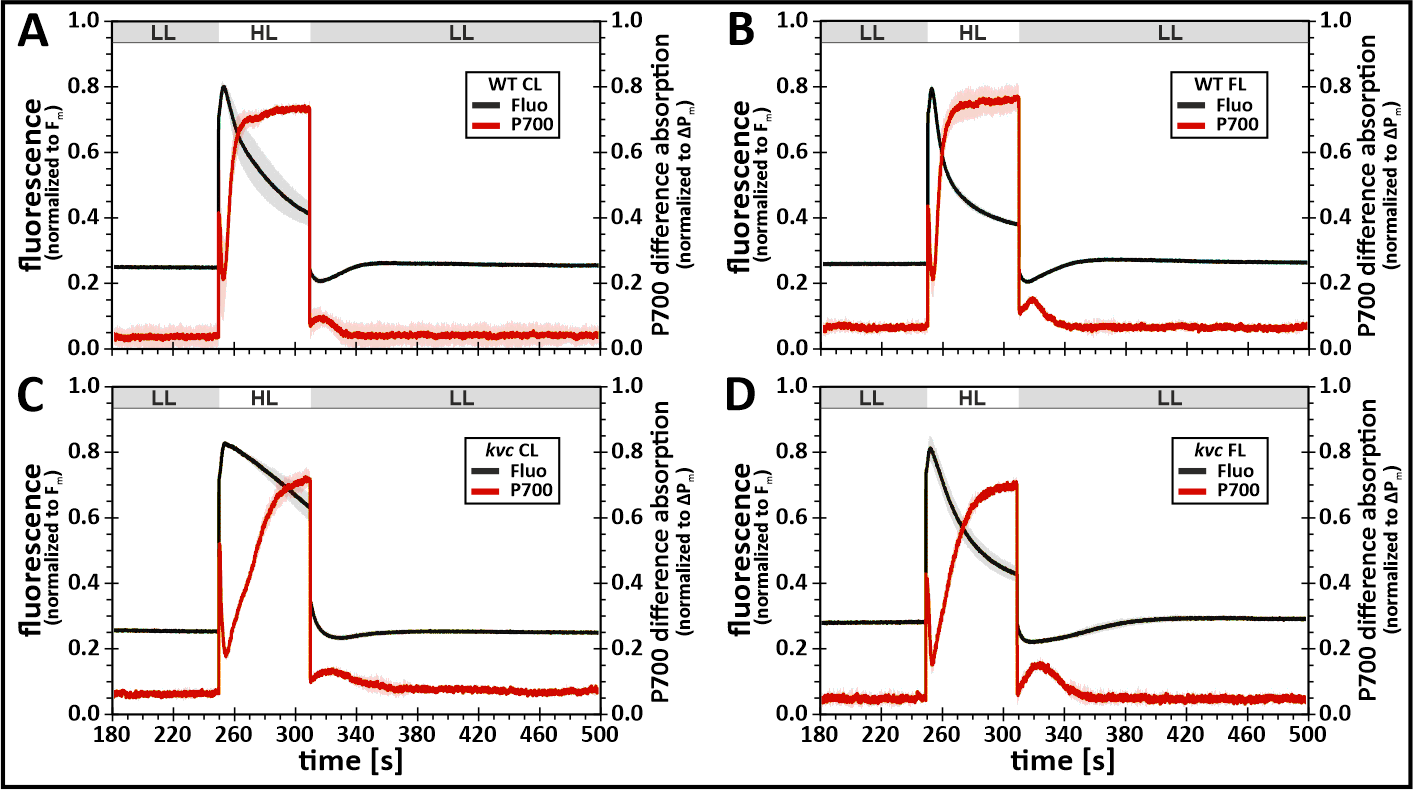


**Supplemental Figure S3: Fluo and P700 traces of WT and *kvc* after CL and FL treatment.**Wild type (WT; **A**,**B**) and *kvc* triple mutant (*kvc*; **C**,**D**) plants were treated for 6 h constant light (CL, 100 µmol photons m^-2^ s^-1^; **A,C**) or 6 h fluctuating light (FL, 50 µmol photons m^-2^ s^-1^ for 4 min and 500 µmol photons m^-2^ s^-1^ for 1 min; **B,D**) immediately prior measurements and subjected to a single FL cycle without dark-acclimation (for details see Methods). Chlorophyll*-a* fluorescence (Fluo, black) and P700 difference absorption (P700, red) traces were recorded with a Dual-PAM 100 system using a single FL cycle indicated by the grey (low light (LL), 50 µmol photons m^-2^ s^-1^) and white bars (high light (HL), 500 µmol photons m^-2^ s^-1^) at the top of each image. Data represents mean with shading above/below the curves indicating standard deviation among replicates (n = 3-4).


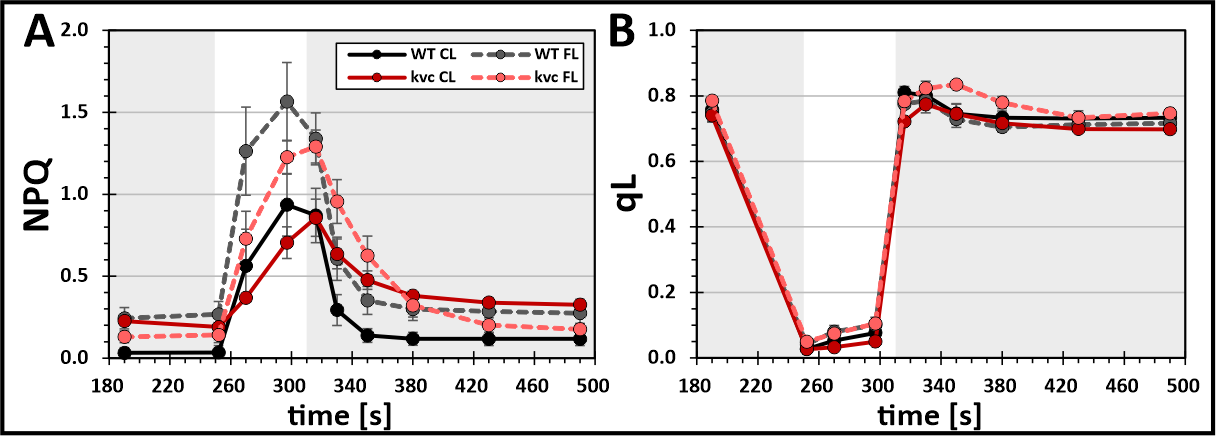


**Supplemental Figure S4: Changes of NPQ and qL in WT and *kvc* after CL and FL treatment.**Wild type (WT - grey) and *kvc* triple mutant (*kvc* - red) were treated for 6 h constant light (CL, 100 µmol photons m^-2^ s^-1^) or 6 h fluctuating light (FL, 50 µmol photons m^-2^ s^-1^ for 4 min and 500 µmol photons m^-2^ s^-1^ for 1 min) immediately prior to measurements with a Dual-PAM 100 system and subjected to a single FL cycle without dark-acclimation (for details see Material and Methods). (**A**) rate constant of non-photochemical quenching (NPQ) in WT and *kvc* after CL and FL treatment; (**B**) photochemical quenching parameter qL, estimating the fraction of open and functional PSII centres, in WT and *kvc* after CL and FL treatment. Data represents mean with error bars indicating standard deviation (n = 3-4).
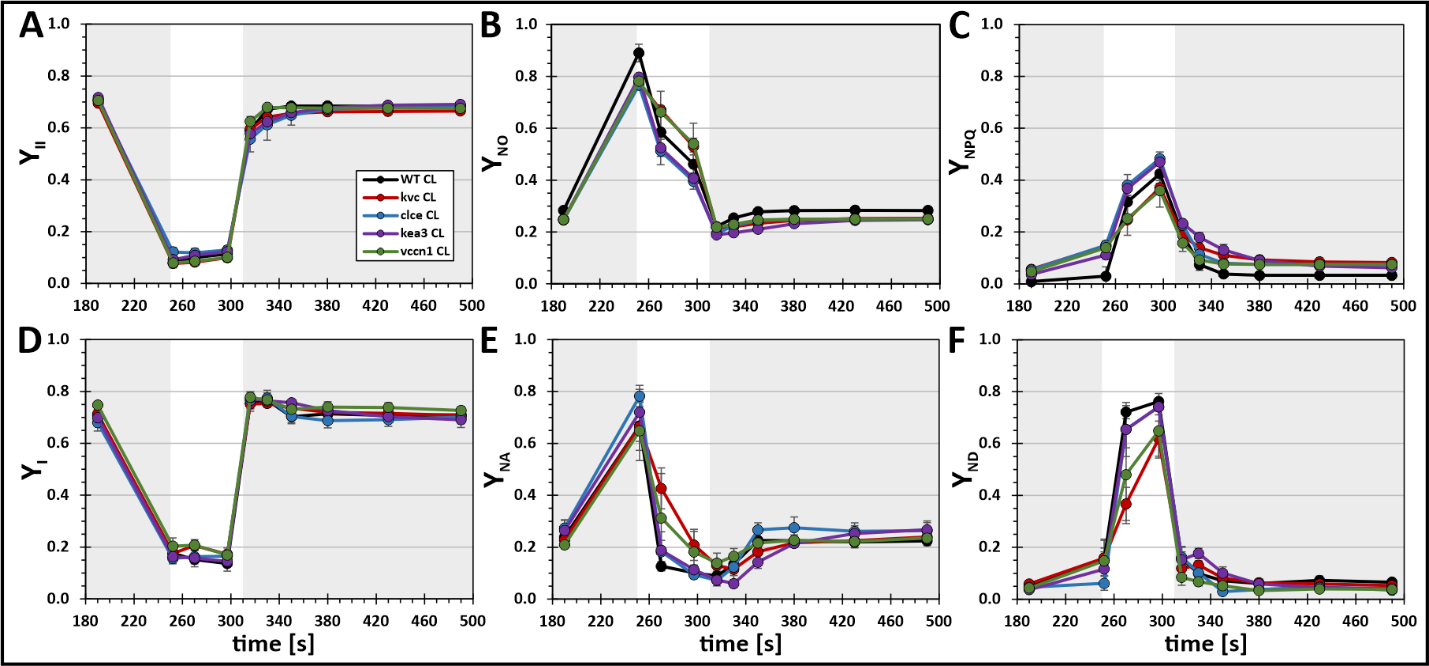


**Supplemental Figure S5: Changes in PSII and PSI quantum yields in WT, *kvc* triple mutant and *clce*, *kea3* and *vccn1* single mutants after CL treatment.**

Wild type (WT, black), *kvc* triple mutant (*kvc*, red), *clce* single mutant (*clce*, blue), *kea3* single mutant (*kea3*, purple) and *vccn1* single mutant (*vccn1*, green) plants were treated for 6 h with constant light (CL, 100 µmol photons m^-2^ s^-1^) immediately prior to measurements with a Dual-PAM 100 system and subjected to a single FL cycle without dark-acclimation (for details see Methods). (A) effective quantum yield of photochemistry in PSII (Y_II_); (B) quantum yield of non-regulated energy dissipation (Y_NO_); (C) quantum yield of regulated non-photochemical quenching (Y_NPQ_); (D) effective quantum yield of photochemistry in PSI (Y_I_); (E) yield of PSI acceptor-side limitation (Y_NA_); (F) yield of PSI donor-side limitation (Y_ND_). Data represents mean with error bars indicating standard deviation (n = 3-4).


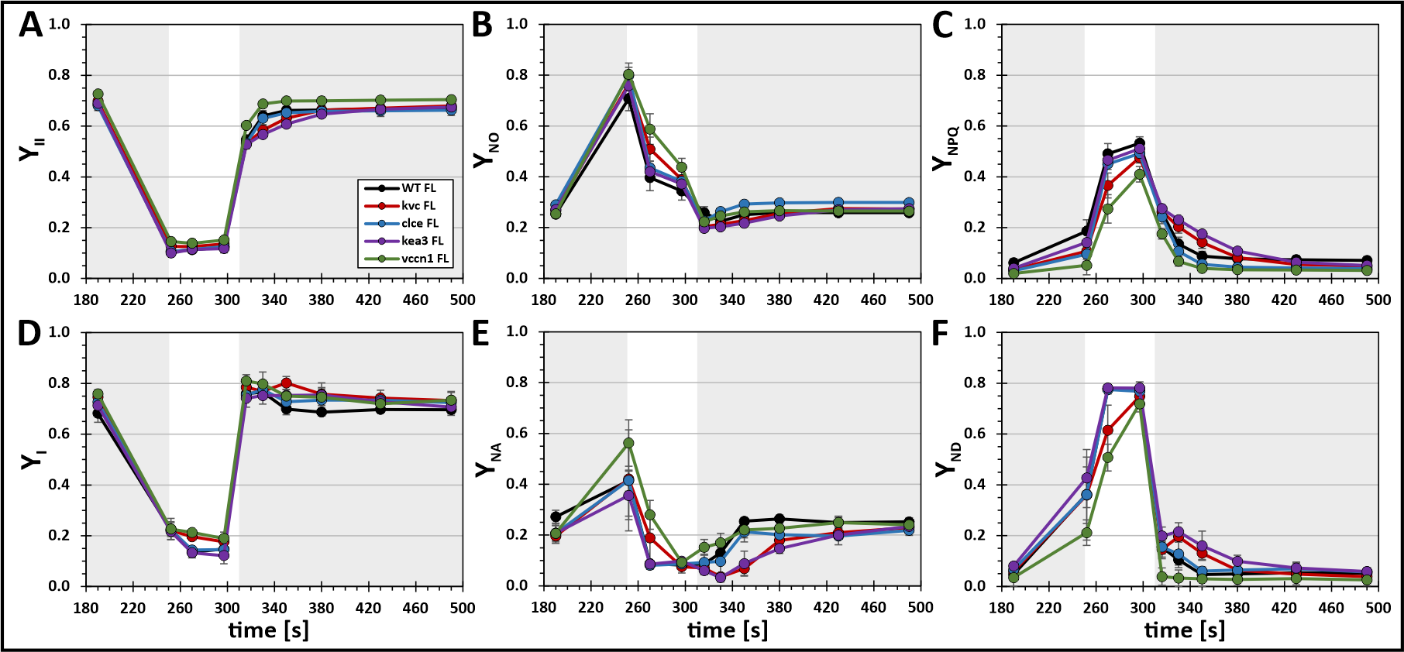


**Supplemental Figure S6: Changes in PSII and PSI quantum yields in WT, *kvc* triple mutant and *clce*, *kea3* and *vccn1* single mutants after FL treatment.**

Wild type (WT, black), *kvc* triple mutant (*kvc*, red), *clce* single mutant (*clce*, blue), *kea3* single mutant (*kea3*, purple) and *vccn1* single mutant (*vccn1*, green) plants were treated for 6 h with fluctuating light (FL, 50 µmol photons m-2 s-1 for 4 min and 500 µmol photons m-2 s-1 for 1 min) immediately prior to measurements with a Dual-PAM 100 system and subjected to a single FL cycle without dark-acclimation (for details see Methods). (A) effective quantum yield of photochemistry in PSII (Y_II_); (B) quantum yield of non-regulated energy dissipation (Y_NO_); (C) quantum yield of regulated non-photochemical quenching (Y_NPQ_); (D) effective quantum yield of photochemistry in PSI (Y_I_); (E) yield of PSI acceptor-side limitation (Y_NA_); (F) yield of PSI donor-side limitation (Y_ND_). Data represents mean with error bars indicating standard deviation (n = 3-4).


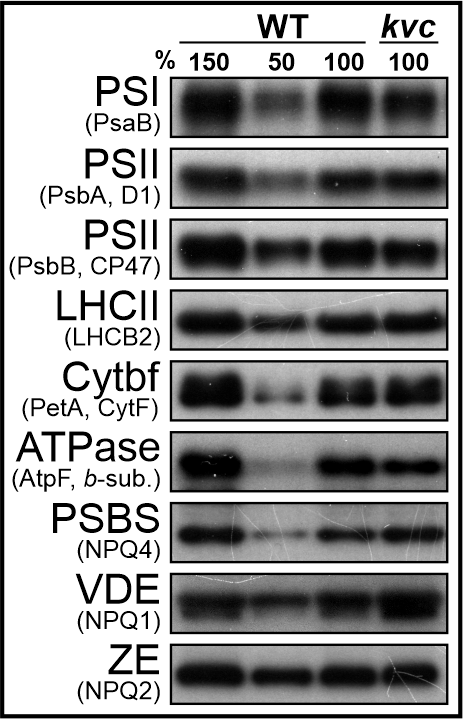


**Supplemental Figure S7: Changes of thylakoid protein abundance in WT and *kvc* grown in FL conditions.**Representative western blots from thylakoid isolation from wild type (WT) and *kvc* triple mutant (*kvc*) grown in FL conditions (FL, 50 µmol photons m^-2^ s^-1^ for 4 min and 500 µmol photons m^-2^ s^-1^ for 1 min) for 6 weeks. Dilution series (150 and 50%) from WT sample.

**
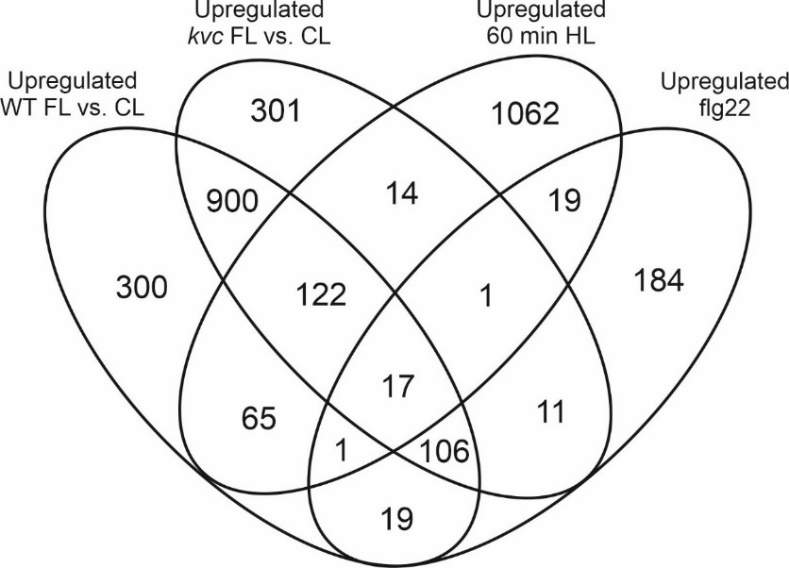
**

**Supplemental Figure S8: Common and distinct expression profiles induced by fluctuating light and other stresses.**Venn diagram illustrating the overlap between genes upregulated by fluctuating light (FL) in wild type (WT) or the *kvc* triple mutant, compared to constant light (CL), genes up regulated by 60 min of high light (HL) treatment (taken from Crisp et al 2017), and genes up regulated by flg22 treatment (taken from Qutob et al., 2006).


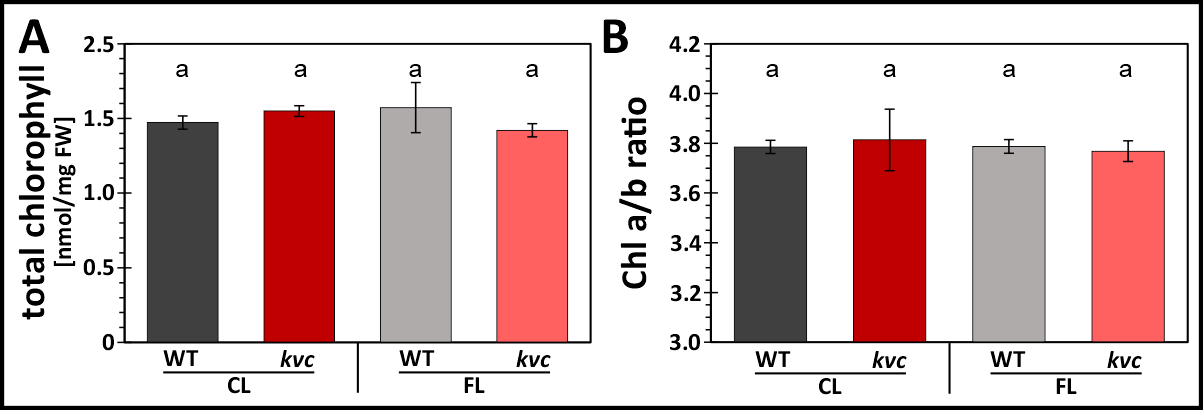


**Supplemental Figure S9: Total chlorophyll content and chlorophyll a/b ratio**(**A**) Total abundance of chlorophyll (Chl) in WT and *kvc* leaves exposed to CL or FL. (**B**) Chlorophyll a/b ratio in WT and *kvc* leaves exposed to CL or FL. Data represents mean with letters indicating statistically significant groups (ANOVA, Tukey-HSD, P<0.05, error bars denote SD, n=5).

**Supplemental data File 1**

Sheet 1: All genes differentially expressed (FC≥ log2 2, FC ≤ log2-2, FDR-corrected p < 0.05) in fluctuating light (FL) vs. constant light (CL) in either wild type (WT) or *kvc* samples

Sheet 2: All genes differentially expressed (FC≥ log2 2, FC ≤ log2-2, p < 0.05) in *kvc* mutant vs wild type (WT) after 6 h constant light (CL) exposure

Sheet 3: All genes differentially expressed (FC≥ log2 2, FC ≤ log2-2, p < 0.05) in *kvc* mutant vs wild type (WT) after 6 h fluctuating light (FL) exposure

**Supplemental data File 2**

Full and non-redundant lists of gene ontology (GO) terms (biological process) enriched in genes upregulated after 6 h fluctuating light (FL) exposure vs. 6 h constant light (CL) exposure in wild type (WT; Sheet 1) or *kvc* (Sheet 2), or in *kvc* vs. WT after 6 h CL exposure (Sheet 3).
