# Supplementary material for: Photosynthetic and transcriptome responses to fluctuating light in Arabidopsis thylakoid ion transport triple mutant
Source: Plant Direct. 2023 Oct 25;7(10):e534. doi: 10.1002/pld3.534 (PMC10598627; doi:10.1002/pld3.534)
Supplement: Supplementary file 5 — Data S5. Peer Review. [file PLD3-7-e534-s002.docx]

**DECISION LETTER – Round 1**

June 2, 2023

Prof. Dr. Eva-Mari Aro
University of Turku
Department of Life Technologies
Department of Biology, Plant Physiology and Molecular Biology
FIN-20014 Turku 20014
Finland


RE: Photosynthetic and transcriptome responses to fluctuating light in Arabidopsis thylakoid ion transport triple mutant

Dear Dr. Aro:

Thank you for submitting to Plant Direct. All required reviews have been returned and we have now finished our evaluation of your manuscript. In light of the reviewers' and editor's comments, minor revisions are needed before the paper can be accepted for publication in Plant Direct.

Please view the editors' and reviewers' comments below and use their suggestions as a guide while you work on your revision.

When uploading the revised version of this article, please be sure to include the following:

-A word document that contains your response to the reviewers. You should respond to each reviewer comment and note the changes made to the manuscript. If you do not agree with a reviewer's comment and choose not to make a suggested revision, please explain why. Please try to provide as complete an answer as possible to each reviewer's criticisms.
-A tracked changes document with each change highlighted
- A clean version of the latest version of the manuscript


To upload your revision, please click the link below.
https://plantdirect.msubmit.net/cgi-bin/main.plex?el=A1Lr7aC1A7au1I5A9ftdR5OKT9AzlxFD6vSqdwQbkwZ


In order to provide as timely a service as possible, we ask that your revision is resubmitted within three months after receipt of this request. If an extension is needed, please send a request, along with a brief explanation, to the editorial office at plantdirect@wiley.com .

Please note that, in addition to publishing reviewer comments, the author's responses to review comments will also be published alongside the final version of the paper. If you would not like the author's responses to be published, please contact the editorial office at plantdirect@wiley.com .


Thank you very much for giving us an opportunity to review your work. I look forward to receiving the next version.


Sincerely,

Yan Lu

Larry York

Editor, Plant Direct


---------------------------------------------------------------------------- Reviewer comments:
Reviewer #1:

Summary:
The manuscript entitled "Photosynthetic and transcriptome responses to fluctuating light in Arabidopsis thylakoid ion transport triple mutant" provides some further insights into the collective function of the known thylakoid ion transport proteins VCCN1, CLCe and KEA3 in response to fluctuating light. My main concern is the lack of a discussion of previous results on light acclimation and integration with the new data. This should be adjusted before publication.
Main points:
Generally, it would have been preferable to also have data on the single mutants. I do not fully understand the rationale of only using the triple mutant. Information on the contribution of single ion transport proteins are thus missing.
-Introduction
There should be an introduction of light acclimation responses. Multiple recent papers have shown that growth light conditions strongly affect photosynthesis and photoprotection. This is of importance for the current manuscript, as different lengths of fluctuating light treatments were used.
-Results
Ln 198 Replace minute with small? Is a bit confusing with the time scales of fluctuations being in the minute range
Ln 189-243 Very descriptive. May help the understanding, if results were shortly summarized and put into context at the end of the paragraph

Ln 246-247 "as short term FL‐treatments typically do not lead to changes in protein contents." Please provide citation or other evidence supporting this statement.


Ln 257-258 Check previous analyses of WT from the Matsubara lab and compare. There is a time of the day effect that interacts with fluctuating light. Which time of the photoperiod were samples taken, when were plants shifted?

Ln 312- Impact of FL on xanthophylls was shown before in the context of a loss of kea3 and longer FL treatments (von Bismarck et al. 2023)

-Discussion

Ln 415-420 "It is unlikely that changes in protein contents would occur upon the short FL treatment and would be directly responsible for rapid changes in the NPQ response, but rather due to transient decrease in lumen pH during the HL phase of the FL‐treatment, explaining the observed higher accumulation of de epoxidized xanthophylls after FL‐treatment in both WT and kvc (Figure 4A,B) (Alter et al., 2012; Wei et al., 2021)." Please fix sentence. The relative clause, starting at ".., but" does not connect with the main clause and is difficult to understand.

Ln 440-445 Sentences are long and difficult to understand. Why would ATP synthase be specifically down in kvc? Don't get the argumentation, which would suggest the same for WT. Cited publication (Wei et al) refers to rice..


Reviewer #2:

In this study, Gollan et al. authors examined the functional role of photosynthetic energy balance in regulating light reactions, gene expression patterns and metabolisms by comparing the Arabidopsis triple mutant lacking KEA3, VCCN1 and CLCe (kvc) with WT under fluctuating light. They found that markedly increased photoprotective energy dissipation of both photosystems and also moderate PSI photoinhibition in kvc. Strikingly, FL induced gene expression changes associated not with primary metabolism, but with secondary metabolism and biotic stress that may be due to cell signaling. The results presented support the main findings here, and I have only minor points before acceptance.

1. Different levels of significance for the comparison between WT and kvc mutant plants under CL and FL can be shown directly in the relevant Figures (including Supplemental Figures) or described in the legends, which make readers more easily and quickly to get information, though the authors presented some in the main text.
2. Did the kvc mutant show any visible growth phenotypes compared to WT in long‐term FL growth conditions? Given that a decrease in PSI (PsaB), ATP synthase (AtpF) was seen in kvc mutant, it is curious to know if this decrease would reflect growth under long‐term FL treatment. In addition, genes involved in biotic stress and defense responses were upregulated despite in short‐term 6h FL, in the long term FL, if these responses would also affect plant growth.

--

DECISION LETTER- Round 2

August 30, 2023
Prof. Dr. Eva-Mari Aro
University of Turku
Department of Life Technologies
Department of Biology, Plant Physiology and Molecular Biology
FIN-20014 Turku 20014
Finland


MSID: 2023-01157-TWR1
MS TITLE: Photosynthetic and transcriptome responses to fluctuating light in Arabidopsis thylakoid ion transport triple mutant

Dear Dr. Eva-Mari Aro:

I am pleased to inform you that your manuscript "Photosynthetic and transcriptome responses to fluctuating light in Arabidopsis thylakoid ion transport triple mutant" has been accepted for publication in Plant Direct.

Your article will appear online in the next available issue of Plant Direct. To ensure your article gets published as quickly as possible, please pay attention to the steps detailed below. We have found that most of the delays happen at this stage, especially at the payment stage, so please respond as quickly as possible when prompted.

License Agreement: Once your article has been accepted it will move to Production and undergo admin and file checking - you may receive an email with any queries we have at this stage. When all required items are received by the publisher and queries resolved, the corresponding author will receive an email from Wiley's Author Services system which will ask them to log on at https://authorservices.wiley.com/bauthor and will present them with the appropriate license for completion. Your article cannot be published until both the signed license agreement and payment of the article fee have been received.

Payment of the Open Access Article Publication Fee: All articles published in Plant Direct are fully open access: immediately and freely available to read, download and share. Plant Direct charges a publication fee to cover the publication costs. The corresponding author for this manuscript should have already received a quote from the payments team (cs-openaccess@wiley.com) with the estimated article publication fee; please email cs-openaccess@wiley.com if this has not been received. The corresponding author should log on to the Wiley Author Services site, where the publication fee can be paid by credit card or an invoice. Pro Forma can also be requested. Payment of the publication charge must be received before the article will be published online.

Proofs: You will have the opportunity to look over your paper once more when you receive the author proofs for your article. The proofs will be with you in approximately two weeks. Please note that, in addition to publishing reviewer comments, the author's responses to review comments will also be published alongside the final version of the paper. If you would not like the author's responses to be published, please contact the editorial office at plantdirect@wiley.com .

Promotion of your article: You can help your research get the attention it deserves! Check out Wiley's free Promotion Guide for best-practice recommendations for promoting your work at www.wileyauthors.com/eeo/guide. And learn more about Wiley Editing Services which offers professional video, design, and writing services to create shareable video abstracts, infographics, conference posters, lay summaries, and research news stories for your research at www.wileyauthors.com/eeo/promotion.

Thank you again for your contribution to Plant Direct. If you have any questions, feel free to contact the editorial office at plantdirect@wiley.com .

Sincerely,

Yan Lu

Larry York

Editor, Plant Direct


---------------------------------------------------------------------------- Editor comments


---------------------------------------------------------------------------- Reviewer comments:

**AUTHORS’ RESPONSE**

**Detailed responses to reviewers**

**Reviewer #1:**
The manuscript entitled "Photosynthetic and transcriptome responses to fluctuating light in Arabidopsis thylakoid ion transport triple mutant" provides some further insights into the collective function of the known thylakoid ion transport proteins VCCN1, CLCe and KEA3 in response to fluctuating light. My main concern is the lack of a discussion of previous results on light acclimation and integration with the new data. This should be adjusted before publication.

**Detailed response to concerns of Reviewer #1:
Concern 1)** Generally, it would have been preferable to also have data on the single mutants. I do not fully understand the rationale of only using the triple mutant. Information on the contribution of single ion transport proteins are thus missing.
**OUR RESPONSE:** The main reason for the focus on the *kvc* triple mutant in the current manuscript were the overall similar responses of *kvc* and single mutants *clce*, *kea3* and *vccn1*. Both the *in-vivo* PSII and PSI parameters as well as differential gene expression showed similar results after short-term FL-treatment. The results of single mutants are now added in the new **Supplemental Tables S1-2** and **new Supplemental Figures (S2, S5, S6 – new numbering)**. Additionally, we have amended the different sections of the main text:

- Material and Methods section mentions individual genotypes, which were used for experiments **(L 102, 114, 163, 173, 182, 188)**, in addition to various other places in results and discussion.
- Results and Discussion mention similar reduction of F_v_/F_m_ and slightly less pronounced reduction of ΔP_m_ **(L 207-210, new Supplemental Figure S2)** as well as similar responses of PSII and PSI quantum yields **(L 252-256, L 373-374, L 406-407, new Supplemental Figure S5 and S6)** in single mutants compared to *kvc* in response to the short-term FL-treatment.
- Results and Discussion mention similar upregulation of genes involved in secondary metabolism between WT and *kvc* as well as single mutants after short-term FL-treatment in comparison to CL-treatment **(L 332-335, L 493-496, new Supplemental Table S1)**.
- The addition of single mutants did not change the conclusions previously based only on *kvc.*
  - However, because single mutants showed slightly less PSI photoinhibition, we have added a possibility of altered ion-homeostasis as cause for slightly enhanced PSI photoinhibition in *kvc* **(L 428-435)**, which should be further investigated.
  - Furthermore, *clce* and *vccn1*, but not *kea3*, showed upregulation of biotic stress response already in (non-stress) CL-conditions, similar to *kvc* **(L 335-339, new Supplemental Table S1)**, which suggests that biotic stress response signaling might be linked to altered thylakoid Cl^-^ ion-homeostasis **(L 531-534)**, which should be further investigated.

**Concern 2)** There should be an introduction of light acclimation responses. Multiple recent papers have shown that growth light conditions strongly affect photosynthesis and photoprotection. This is of importance for the current manuscript, as different lengths of fluctuating light treatments were used.
**OUR RESPONSE:** Thank you for pointing out this oversight, we have modified the introduction **(L 48-54)** to mention different light acclimation responses supported by recent literature. However, we have not added more details regarding this topic, because they largely overlap with already provided information about photoprotective mechanisms, regulation of ion-channels and altered gene expression in the introduction. These are most relevant for the short-term FL-response (6h) that was the main focus of this study. The only exception was when *kvc* plants were grown in FL for 6 weeks to validate the long-term effects of PSI photoinhibition on degradation and re-organisation of key photosynthetic thylakoid proteins. This was necessary, since short-term FL treatments used in this study, are not expected to be majorly affected on the protein level (for references, see also response to Concern 5). To accommodate for differences in the time points of the FL acclimation response, we have modified the discussion of the PSI photoinhibition **(L 417-427)** pointing out difficulties dealing with different lengths of FL treatments as well as the dependency of PSI photoinhibition on the applied FL-conditions (Kono and Terashima 2014; Journal of Photochemistry and Photobiology B: Biology; Sejima et al. 2014, Plant Physiology; Tikkanen and Grebe 2018, Physiologia Plantarum; Tan et al. 2021, Environmental and Experimental Botany).

**Concern 3)** Ln 198 Replace minute with small? Is a bit confusing with the time scales of fluctuations being in the minute range.
**OUR RESPONSE:** We have replaced “minute” with “small” **(L 204)**.

**Concern 4)** Ln 189-243 Very descriptive. May help the understanding, if results were shortly summarized and put into context at the end of the paragraph.
**OUR RESPONSE:** Thank you for pointing this out. We have summarized the results of this section at the end of the paragraph **(L 265-268)**.

**Concern 5)** Ln 246-247 "as short-term FL‐treatments typically do not lead to changes in protein contents." Please provide citation or other evidence supporting this statement.
**OUR RESPONSE:** Thank you for pointing out this overgeneralization. We have adjusted the results section to better reflect the literature **(L 263-270)**. Short-term changes typically do not lead to major (not none) adjustments at the protein level (Schöttler and Tóth 2014, Frontiers in Plant Science; Kono and Terashima 2014, Journal of Photochemistry and Photobiology B: Biology) in contrast to long-term acclimation (Niedermaier et al. 2020, Frontiers in Genetics; Flannery et al. 2021, Plant Journal). Since long-term FL-experiments were specifically targeted to investigate sensitivity of *kvc* to PSI photoinhibition, we also included further explanations on why photosystem protein abundances are unlike to change in short-term experiments. These relate to the activity of the PSII repair cycle (Aro et al. 1993, Biochimica et Biophysica Acta – Bioenergetics; Järvi et al. 2015, Biochimica et Biophysica Acta – Bioenergetics) and high stability of PSI reaction centers despite PSI photoinhibition (Kudoh and Sonoike, 2002, Planta; Zhang and Scheller 2004, Plant Cell & Physiology; Tiwari et al. 2016, Nature Plants; Lempiäinen et al. 2022, Plant Cell & Environment).

**Concern 6)** Ln 257-258 Check previous analyses of WT from the Matsubara lab and compare. There is a time of the day effect that interacts with fluctuating light. Which time of the photoperiod were samples taken, when were plants shifted?
**OUR RESPONSE:** Thank you for pointing out this missing information. Plants were shifted to CL- or FL-conditions at the beginning of the photoperiod so that sample collection was always performed towards end of the photoperiod. We have added this clarification in the Material and Methods **(L108-113)** and refer to recent study from the Matsubara lab (Schneider et al. 2019, Plant Physiology) for time of day effects.

**Concern 7)** Ln 312- Impact of FL on xanthophylls was shown before in the context of a loss of kea3 and longer FL treatments (von Bismarck et al. 2023).
**OUR RESPONSE:** We have added the Bismarck et al 2023 reference to the relevant section in the discussion **(L 464-465).**

**Concern 8)** Ln 415-420 "It is unlikely that changes in protein contents would occur upon the short FL treatment and would be directly responsible for rapid changes in the NPQ response, but rather due to transient decrease in lumen pH during the HL phase of the FL‐treatment, explaining the observed higher accumulation of de epoxidized xanthophylls after FL‐treatment in both WT and kvc (Figure 4A,B) (Alter et al., 2012; Wei et al., 2021)." Please fix sentence. The relative clause, starting at ".., but" does not connect with the main clause and is difficult to understand.
**OUR RESPONSE:** Thank you for pointing this out, we have modified the section to be better understandable **(L 459-465)**.

**Concern 9)** Ln 440-445 Sentences are long and difficult to understand. Why would ATP synthase be specifically down in kvc? Don't get the argumentation, which would suggest the same for WT. Cited publication (Wei et al) refers to rice.
**OUR RESPONSE:** We acknowledge that this section was not clearly written and have modified it **(L 490-493).** In general, a decrease of ATP synthase content has been associated with increase in PMF (e.g. Rott et al. 2011, Plant Cell; Kanazawa et al. 2017, Frontiers in Plant Science). Since *kvc* lacks the possibility to modulate PMF via ion-channels/transporters, it seems plausible that the long-term FL-acclimation strategy might involve an overall decrease of ATP synthase content. This could help *kvc* to enhance PMF during illumination and consequently enhance the NPQ response dependent on PMF, despite the lack of ion-channels/transporters. A similar response is not expected for WT, because WT retains the ability to modulate PMF and NPQ via thylakoid ion-channels/transporters. However, we do not see an issue with results in rice from Wei et al 2021, since it can be assumed that photosynthetic responses to FL between different angiosperms are generally comparable.

**Reviewer #2:**

In this study, Gollan et al. authors examined the functional role of photosynthetic energy balance in regulating light reactions, gene expression patterns and metabolisms by comparing the Arabidopsis triple mutant lacking KEA3, VCCN1 and CLCe (kvc) with WT under fluctuating light. They found that markedly increased photoprotective energy dissipation of both photosystems and also moderate PSI photoinhibition in kvc. Strikingly, FL induced gene expression changes associated not with primary metabolism, but with secondary metabolism and biotic stress that may be due to cell signaling. The results presented support the main findings here, and I have only minor points before acceptance.

**Detailed response to concerns of Reviewer #2:**

**Concern 1)** Different levels of significance for the comparison between WT and kvc mutant plants under CL and FL can be shown directly in the relevant Figures (including Supplemental Figures) or described in the legends, which make readers more easily and quickly to get information, though the authors presented some in the main text.
**OUR RESPONSE:** Thank you for this suggestion, we have modified the **main figures 1, 4 and 5 as well as supplemental figures S2 and S9** **(new numbering)** with significance levels, updated Figure legends **(L 594-596, 627-628, 631-632)** and accordingly removed individual t-tests from the results section. Additionally, we updated individual t-tests to more appropriate oneway-ANOVAs, which did not affect the significant levels. Because of unequal variance between samples in the cuticle permeability experiment (**main figure 6**), we have kept the t-test results based on two-sample unequal variance t-tests and modified the figure to show significance levels accordingly.

**Concern 2)** Did the kvc mutant show any visible growth phenotypes compared to WT in long‐term FL growth conditions? Given that a decrease in PSI (PsaB), ATP synthase (AtpF) was seen in *kvc* mutant, it is curious to know if this decrease would reflect growth under long‐term FL treatment. In addition, genes involved in biotic stress and defense responses were upregulated despite in short‐term 6h FL, in the long-term FL, if these responses would also affect plant growth.
**OUR RESPONSE:** Unfortunately, we did not collect data to investigate differences in growth between genotypes. However, based purely on visual observation, the *kvc* or single mutants did not show a noticeable growth phenotype compared to WT in either short- or long-term FL treatments.
With regards to biotic stress and defense responses upregulated in *kvc* (as well as *clce* and *vccn1*, **new Supplemental Table S1**) already in CL-conditions, previous work did not find any significant effect on plant growth under constant light conditions after 5 weeks (see Fig. 1, Dukic et al. 2019, Scientific Reports).
Regarding the PSI and ATP synthase protein contents, previous work on tobacco has established that photosynthetic and growth phenotypes require reductions of >50% at the protein level, as both proteins appear to be present in large excess amounts in the thylakoid membrane (see Schöttler and Tóth 2014, Frontiers in Plant Science and references therein).
